# The Paradox of Workforce Self‐Sufficiency: Policy Narratives, Professional Identity and Internationally Educated Nurses in the United Kingdom

**DOI:** 10.1111/nin.70164

**Published:** 2026-09-08

**Authors:** Joshua Chidiebere Nwambo, Oladayo Bifarin, Karen Higginbotham

**Affiliations:** ^1^ School of Nursing, Public and Allied Health Liverpool John Moores University Liverpool UK

**Keywords:** conditional belonging, internationally educated nurses, NHS Long Term Workforce Plan, nursing leadership, policy analysis, professional identity, qualitative evidence synthesis, workforce governance

## Abstract

The NHS Long Term Workforce Plan positions international recruitment as essential for present stability while projecting a future of reduced structural dependence on internationally educated nurses. This tension, conceptualised as the paradox of self‐sufficiency, forms the focus of this study. Drawing on a critical qualitative evidence synthesis, eight UK policy documents were analysed using Bacchi's ‘What's the Problem Represented to Be?’ (WPR) framework alongside thematic synthesis of 17 qualitative studies, sensitised by Social Identity Theory and Ontological Security. Policy analysis reveals that the Plan positions international recruitment as a short‐term necessity, treating domestic training as the preferred long‐term pathway while leaving internationally educated nurses uncertain about their long‐term place within the workforce. Three consistent patterns emerge from the qualitative evidence: prior expertise requires institutional revalidation before professional legitimacy is accorded; the burden of professional reconstruction falls disproportionately on internationally educated nurses, and belonging remains conditional rather than secure. These patterns are not incidental but are suggestive of underlying structural conditions mediated through organisational contexts but cannot be resolved by organisations alone. Sustainable workforce planning requires policy‐makers and nursing leadership to recognise internationally educated nurses as permanent contributors, ensure equitable career development and build organisational cultures in which belonging is unconditional.

## 1 Introduction

The United Kingdom's National Health Service (NHS) has historically relied on internationally educated nurses (IENs) to address persistent workforce shortages. From post‐war recruitment across Commonwealth countries to sustained inflows from the Philippines, India and Nigeria, internationally trained professionals have remained integral to NHS service delivery (Buchan et al. 2019; Mpando et al. 2025). As of 2025, approximately one quarter of professionals on the Nursing and Midwifery Council (NMC) register were internationally educated, illustrating the continuing importance of global labour mobility to workforce stability (Nursing and Midwifery Council 2025).

A significant shift in workforce strategy occurred with the publication of the NHS Long Term Workforce Plan (LTWP) in 2023, the first comprehensive 15‐year workforce strategy in NHS history (NHS England 2023). The Plan sets out a substantial change in how the NHS intends to build and sustain its workforce, with consequences that extend beyond staffing numbers to how different parts of the workforce are positioned within long‐term planning. Yet despite its strategic scope, the implications of the LTWP for IENs already embedded within the workforce remain an open question.

This matters because workforce policy does not operate solely as a technical instrument of labour supply planning. Rather, policies construct particular understandings of workforce problems and position professional groups in specific ways (Bacchi 2009). These representations shape narratives of legitimacy, belonging and permanence within healthcare systems while reflecting the broader socio‐political climate. Since Brexit, the context in which IENs navigate NHS employment has shifted considerably, raising questions about how workforce strategies interact with already unsettled conditions of professional belonging and settlement among migrant health workers (Spiliopoulos and Timmons 2023). Whether and how the LTWP's approach to international recruitment positions IENs within these conditions, and what that positioning means for their professional identity and belonging, remains underexamined.

Existing research on IENs has primarily examined transitions, workplace integration, belonging and professional identity through organisational, cultural and interpersonal lenses (Alexis and Shillingford 2015; Bond et al. 2020; Joseph et al. 2023; Lanada and Culligan 2024). Less attention has been given to how workforce policy shapes the conditions within which these processes occur. This intersection warrants greater scrutiny given the rapid pace of change in the policy landscape governing NHS workforce planning and international recruitment.

One manifestation of this policy–practice intersection is the growing investment in organisational integration programmes, including induction schemes and pastoral support initiatives, intended to support the recruitment, integration and retention of IENs (Mpando et al. 2025; NHS England 2022, n.d.; Pressley et al. 2023). While these initiatives represent a meaningful organisational response to the challenges IENs face, how they interact with the broader policy environment in which they are situated remains poorly understood (Lanada and Culligan 2024; Rajpoot et al. 2024; Omiyi et al. 2025). Examining this relationship is therefore important for understanding what organisations can realistically achieve in supporting IEN professional identity and belonging.

These gaps carry direct implications for nurse leaders and workforce planners, for whom understanding how national policy and organisational practice interact is critical for sustaining IEN engagement, retention and professional belonging (Mpando et al. 2025; Omiyi et al. 2025). Creating organisational cultures that promote psychological safety, inclusion and participation may be particularly important in supporting these outcomes (Edmondson 2018).

This study examines how the NHS LTWP represents international recruitment within its workforce self‐sufficiency agenda and explores how policy narratives intersect with the professional identity trajectories of IENs. It asks how qualitative accounts of IENs illuminate processes of identity negotiation within the NHS, and how national workforce strategies and organisational integration practices interact to shape leadership and retention challenges.

## 2 Theoretical Framework

This study integrates policy analysis with theories of professional identity to examine how workforce governance narratives intersect with the lived experiences of IENs. Three complementary theoretical perspectives inform the analysis: Bacchi's ‘What's the Problem Represented to Be?’ (WPR) framework, Social Identity Theory and the concept of Ontological Security.

### 2.1 Policy Representation and Workforce Governance

Bacchi's WPR framework provides a critical lens for examining how policy constructs social problems and positions particular groups within governance narratives (Bacchi 2009). Rather than assuming that policies simply respond to pre‐existing problems, the WPR approach emphasises how policy discourse can be understood as constructing a representation through language, assumptions and institutional priorities. These representations shape how a given issue is understood by policy‐makers and institutions, while implicitly positioning affected groups within governance narratives and determining which solutions appear legitimate.

Within workforce planning, policy discourse may frame labour shortages, international recruitment or domestic training capacity as specific kinds of problems requiring targeted interventions. Such framing can also implicitly position internationally educated professionals in particular ways within the healthcare workforce. Applying the WPR framework therefore allows examination of how the NHS LTWP constructs international recruitment within its self‐sufficiency agenda and how these representations shape the symbolic positioning of IENs, building on prior analyses that highlight the discursive governance of migrant health professionals within UK policy contexts (Bond et al. 2020; Spiliopoulos and Timmons 2023).

### 2.2 Social Identity Theory: Group Positioning and Status Differentiation

To understand how such policy representations may influence professional experience, this study draws on Social Identity Theory (Tajfel and Turner 1979). Social Identity Theory proposes that individuals derive part of their self‐concept from membership in social and professional groups. Processes of categorisation, comparison and group positioning influence how individuals interpret belonging, legitimacy and status within organisational contexts.

Concepts central to Social Identity Theory, including group membership, belonging and social positioning, have informed nursing research examining professional hierarchies, intergroup relations and experiences of marginalisation among internationally recruited and ethnic minority nurses (Batnitzky and McDowell 2011; Alexis and Shillingford 2015). In healthcare settings, professional identity is closely linked to recognition of expertise, autonomy and career progression (Johnson et al. 2012; Joseph et al. 2023). When workforce policies differentiate between domestic and IENs, they may be interpreted as creating symbolic distinctions that are relevant when considering perceptions of professional standing. Examining these dynamics provides insight into how IENs negotiate identity and belonging within organisational and policy environments.

### 2.3 Ontological Security and Professional Continuity

The concept of Ontological Security further illuminates the relationship between migration, identity and professional continuity. Ontological Security refers to an individual's sense of stability and continuity in their self‐identity and social environment (Giddens 1991). Migration can disrupt this continuity by requiring individuals to renegotiate professional status, cultural expectations and organisational roles (Alexis and Shillingford 2015).

Previous studies of IENs have used concepts of Ontological Security, belonging and settlement to explain how migration decisions, employment conditions and settlement experiences are closely tied to expectations of long‐term professional and personal stability (Smith and Gillin 2021; Spiliopoulos and Timmons 2023). For IENs, processes such as credential verification, adaptation to new clinical practices, unstable immigration policies and navigation of unfamiliar institutional norms may influence the stability of professional identity. Experiences of conditional belonging or uncertain permanence within host systems may also shape perceptions of professional security. Analysing these dynamics provides insight into how broader workforce governance narratives intersect with individual experiences of identity reconstruction.

### 2.4 Integrating Policy, Identity and Workforce Governance

**Figure 1:**
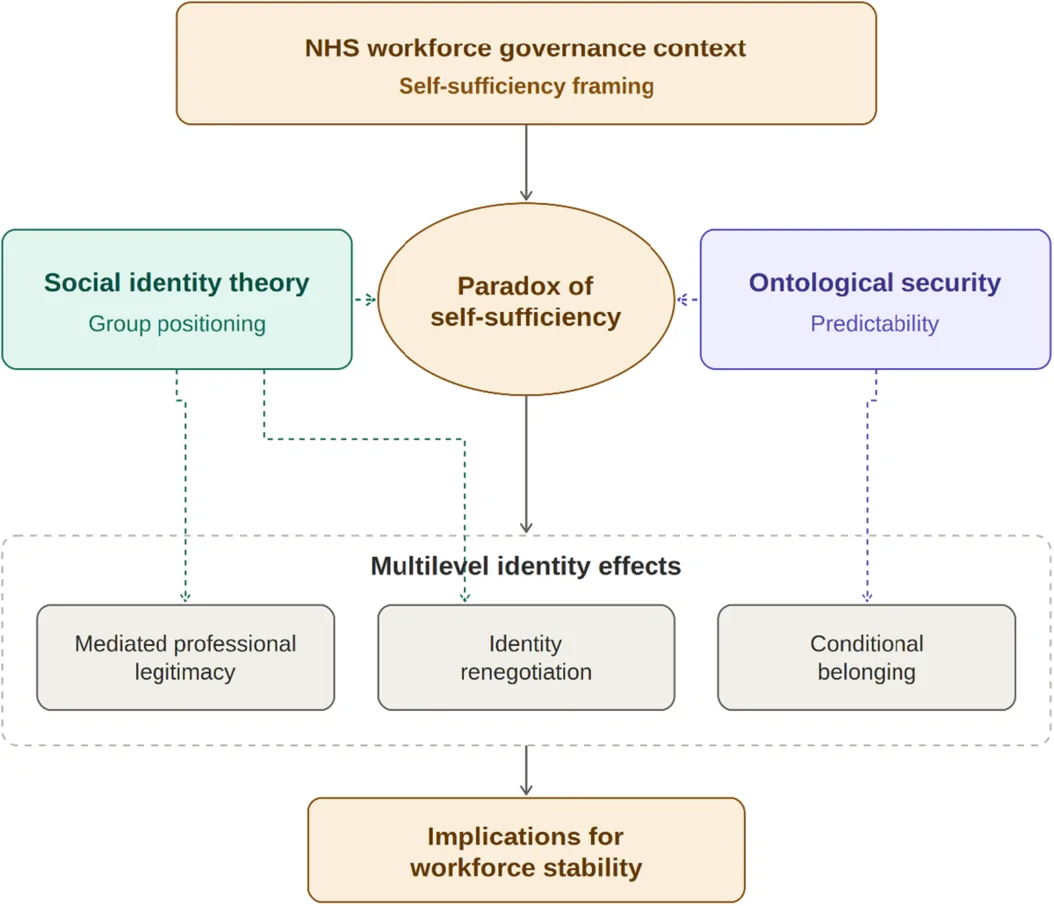
Integrative theoretical framework linking workforce governance and professional identity. Solid arrows indicate directional analytical influence. Dashed arrows represent theoretical lenses applied to the paradox and to the identity effects it produces. Social Identity Theory interprets mediated professional legitimacy and identity renegotiation; Ontological Security interprets conditional belonging. Arrow colour indicates the interpreting lens. The paradox, shaped by NHS workforce governance, converges on three multilevel identity effects, with consequences for workforce stability. Constructs are developed in Section 4.3 and set out in full in Supporting Information S4.

The three frameworks address distinct analytical problems, and their integration is architecturally necessary rather than additive. Together they enable the analysis to move across three dimensions, discursive, social and temporal, that no single framework could address alone. Figure 1 illustrates this analytical architecture and the directional pathways connecting its components.

NHS workforce governance, examined through the WPR framework, is where the paradox of self‐sufficiency takes shape: the simultaneous coexistence of operational indispensability and strategic recalibration towards reduced international recruitment. Social Identity Theory and Ontological Security function as lateral interpretive lenses, applied to the paradox and to the identity effects it produces rather than operating independently. Social Identity Theory illuminates how governance discourse is associated with group‐level effects on professional standing and recognition and is therefore the lens through which mediated professional legitimacy and identity renegotiation are interpreted. Ontological Security illuminates how the same signals intersect with the continuity and predictability upon which stable professional identity depends over time and is accordingly applied to conditional belonging. Together these lenses orient analytical attention towards the multilevel identity effects that follow from the paradox, and towards their consequences for workforce stability, offering an interpretive account of why these patterns recur across diverse organisational contexts.

## 3 Methodology

### 3.1 Study Design

This study employed a critical qualitative evidence synthesis (CQES), integrating policy discourse analysis with secondary qualitative synthesis to produce interpretive findings that extend beyond aggregation into critical explanation (Flemming et al. 2019). Unlike aggregative reviews concerned with pooling evidence to estimate effects, CQES pursues conceptual development and critical interpretation, attending to how evidence is produced, what assumptions it carries and what it renders invisible (Dixon‐Woods et al. 2006; Flemming et al. 2019). This distinction is central to the study's purpose: rather than summarising what is known about IENs' experiences, the review examines how workforce governance narratives shape constructions of professional identity, belonging and legitimacy.

The study integrated two complementary analytical strands: Bacchi's (2009) WPR framework, applied to national policy and regulatory documents, and a thematic synthesis of qualitative empirical literature, applied to UK‐based studies of IENs' professional experiences. Integration of these strands was sequential and subsequently iterative. WPR analysis was conducted first, establishing the analytical horizon, the governance context and its problem representations, against which qualitative evidence was subsequently read. Findings from both strands were then brought into iterative dialogue through constant comparison, enabling identification of convergence, tension and analytical implication across policy and experiential levels.

An abductive analytic logic governed the overall approach, combining inductive sensitivity to patterns emergent within the data with theoretically sensitised interpretation guided by Social Identity Theory and Ontological Security (Timmermans and Tavory 2012). Abductive reasoning permitted movement between the empirical and the theoretical without reducing either to the other, supporting the development of interpretive claims about the intersection of workforce governance and professional identity that could not have been produced by either strand alone.

### 3.2 Policy Selection

Grey literature, comprising policy and governance documents, was not searched through database aggregators. Instead, targeted organisational searches were conducted across key institutions, including NHS England, the Royal College of Nursing, the NMC, The King's Fund, the Nuffield Trust, the Home Office, government white papers and The Health Foundation. This approach was considered appropriate given the stability and centralisation of UK workforce policy, with relevant policy documents consistently produced and made publicly available through a small number of identifiable institutional sources.

Eight policy and statutory documents were purposively selected based on relevance to the regulation, recruitment, governance and integration of IENs within the UK health system, and to capture governance across the macro‐strategic, ethical recruitment, immigration, professional regulatory, equality monitoring and organisational domains. Selection continued until conceptual sufficiency was achieved, defined as the point at which additional documents yielded no new problem representations. A final targeted search was conducted following selection; no additional governance representations that would alter the analytical corpus were encountered, confirming analytical closure.

### 3.3 Data Sources and Search Strategy

A systematic search was conducted across CINAHL, MEDLINE, Scopus and Web of Science to identify qualitative studies examining the experiences of IENs working in the United Kingdom. Searches covered publications from January 2010 to December 2025. The 2010 start point was chosen to capture professional identity negotiation within governance that preceded the formal articulation of workforce self‐sufficiency in the NHS LTWP. Earlier qualitative evidence was therefore treated as historically situated insight into enduring patterns of professional positioning, enabling a diagnostic historical reading of continuity between experiential accounts and later policy framing (Buchan et al. 2019; NHS England 2023).

Search strategies combined controlled vocabulary and keywords related to internationally educated or migrant nurses, the NHS, professional identity, workforce integration and career progression. Studies were eligible if they employed qualitative or mixed‐methods designs with a substantive qualitative component, focused on IENs registered to practise in the United Kingdom and addressed professional experience, identity negotiation, workforce integration or career progression. For mixed‐methods studies, only qualitative data relevant to the review questions were extracted and synthesised. Only peer‐reviewed English‐language publications were included. Studies were excluded if purely quantitative, conducted outside the United Kingdom, focused on non‐registered nursing roles or consisted of commentary or opinion without an empirical basis. Where studies sampled mixed healthcare populations, they were included only if findings attributable to nurses were separately identifiable.

Study selection followed a PRISMA‐informed screening process (Page et al. 2021; see Supporting Information S1) using Rayyan, an AI‐assisted systematic review platform. At title and abstract stage, two reviewers independently screened all records against predefined inclusion criteria, achieving high inter‐rater reliability with 92% raw agreement and a Cohen's kappa of 0.83, indicating almost perfect agreement. Full‐text screening was conducted by one reviewer, with all inclusion decisions subsequently verified by a second independent reviewer; a third reviewer was available to adjudicate unresolved disagreements (Waffenschmidt et al. 2019). Rayyan's conflict detection features supported identification of discordant decisions, which were re‐examined against the eligibility criteria. All discrepancies were resolved through discussion and consensus.

As an additional safeguard, Microsoft Copilot was used to further assess the relevance of each record against the predefined eligibility criteria as a decision‐support check; all final inclusion and exclusion decisions were made by the reviewing authors. Seventeen studies met the inclusion criteria and are presented in Supporting Information S2.

### 3.4 Quality Appraisal

Methodological quality was assessed using the Joanna Briggs Institute (JBI) Critical Appraisal Checklist for Qualitative Research (Joanna Briggs Institute 2020). Scores ranged from 7.5 to 10.0 out of 10, with a mean of 9.5. All 17 studies met the quality threshold for inclusion. The overall quality of the evidence base was uniformly high, and all 17 studies were considered analytically credible contributors to the development of synthetic constructs. No study was excluded on quality grounds, consistent with CQES principles (Dixon‐Woods et al. 2006; Flemming et al. 2019). Study characteristics and full criterion‐level appraisal scores are presented in Supporting Information S2. Two included studies (Pressley et al. 2023, 2025) employed mixed‐methods designs; as only their qualitative findings were extracted and synthesised, these strands were appraised using the JBI Critical Appraisal Checklist for Qualitative Research, consistent with the qualitative focus of this synthesis and with component‐based appraisal in mixed studies reviews (Joanna Briggs Institute 2020).

### 3.5 Data Analysis

Data analysis was conducted across two sequential strands, each described below and subsequently integrated through iterative comparative analysis.

*Strand 1: Policy Discourse Analysis Using the WPR Framework*. Bacchi's (2009) WPR framework was applied systematically to eight policy and regulatory documents. Analysis was structured explicitly around Bacchi's six analytical questions: (1) how the ‘problem’ of workforce sustainability was represented; (2) the assumptions and presuppositions underpinning these representations, including notions of temporality and workforce permanence; (3) the historical, political and discursive conditions through which the representations emerged; (4) what was left unproblematised or silenced; (5) the discursive effects produced, including the subject positions created for IENs, and (6) how the problem could be represented differently.

Each document was read individually and interrogated through Bacchi's six analytical questions, applied flexibly with analytical emphasis weighted according to the salience of particular representations within individual documents. Proposed policy solutions, governance logics and silences were analysed alongside the positioning of IENs within workforce sustainability discourse. Governance framings were compared across documents to identify recurring problem representations rather than isolated policy statements and were classified into eight analytical categories reflecting the distinct governance functions through which IENs' positioning is constructed within the NHS workforce architecture.

This strand generated two direct analytical outputs: (1) an in‐depth WPR analysis of the NHS LTWP (Table 1) as the primary macro‐strategic articulation of workforce self‐sufficiency and (2) a cross‐document analysis of all eight policy instruments (Table 2), mapping the governance layer, strategic framing and operational function through which each instrument positions IENs within the workforce architecture. These findings subsequently informed the integrated analysis of how governance representations are distributed across multiple instruments and manifested in lived professional trajectories.

**Table 1:** WPR analysis of the NHS Long Term Workforce Plan (NHS England 2023).

WPR question	Discursive representation in LTWP (2023)	Indicative evidence from policy text	Analytical implications for IEN professional identity
Q1: What is the problem represented to be?	International recruitment is positioned as a reliance to be reduced in favour of domestic workforce expansion.	‘Reduce reliance on international recruitment’ (p. 8); ‘Dependence on international recruits… scaled back’ (p. 15).	Transitional Positioning: International recruitment is framed as necessary in the short term but not central to long‐term sustainability, potentially shaping perceptions of permanence and belonging.
Q2: What assumptions underlie this representation?	Domestic education and training are constructed as the primary route to workforce sustainability.	‘Putting the workforce on a sustainable footing’ (p. 15); ‘Significantly expanding domestic education’ (p. 7).	Implicit Hierarchisation of Workforce Pathways: Domestic qualification routes are foregrounded as foundational, positioning internationally acquired expertise as supplementary.
Q3: How has this representation come about?	Emphasis on national workforce resilience following pandemic pressures and intensified global labour competition.	Target to reduce international recruitment to ‘9‐10.5%’ (p. 8); references to ‘unprecedented’ pandemic pressures (p. 12).	Conditional Belonging: Policy emphasis on future reduction may create ambiguity regarding long‐term positioning of IENs.
Q4: What is left unproblematised or silent?	Limited engagement with recognition of prior international seniority, specialist skills or career progression pathways.	Focus on retention rates and numerical leavers (pp. 8, 18); minimal reference to IEN‐specific development pathways.	Risk of Professional Discontinuity: Silence regarding expertise recognition may indirectly sustain practices that delay full professional integration.
Q5: What discursive effects are produced?	Distinction between domestic pipeline expansion and continued but reduced international inflow.	Expansion of domestic training alongside acknowledgement of high turnover in certain roles (p. 32).	Differentiated Workforce Positioning: Discursive segmentation may contribute to perceived variation in status and authority across workforce groups.
Q6: How could the problem be represented differently?	International recruitment framed as an enduring and structurally embedded component of a globally interconnected workforce.	international recruitment as a structurally embedded and enduring component of NHS workforce supply, with sustainability defined by retention, progression and stability of the international workforce alongside domestic expansion.	Such reframing may reduce perceptions of conditional permanence, strengthen confidence in long‐term institutional belonging and reinforce continuity of professional identity over time.

*Note:* Policy extracts and page references derived from NHS Long Term Workforce Plan (NHS England 2023). Analytical interpretation informed by Bacchi's (2009) WPR framework.

**Table 2:** Layered policy and governance instruments shaping internationally educated nurses' positioning within NHS workforce governance.

Policy document	Governance layer	Strategic framing	Operational function
NHS Long Term Workforce Plan (2023)	Macro workforce strategy	Workforce self‐sufficiency; domestic expansion	Repositions international recruitment as operationally necessary yet transitional within long‐term supply planning.
Code of Practice for International Recruitment (DHSC 2023)	Ethical recruitment regulation	Managed global sourcing	Regulates ethical recruitment through red/amber country lists, recruiter compliance and organisational accountability.
Health and Care Worker Visa (2020–)	Immigration governance	Skilled migration sponsorship	Links right to reside and settlement pathways to sponsored employment in eligible roles.
NMC Test of Competence (CBT/OSCE) (NMC 2021)	Professional regulation	Competence verification; public protection	Governs overseas entry via CBT/OSCE assessment and employer‐supervised pre‐registration candidacy.
Workforce Race Equality Standard 2023 and 2024 data; reports 2024 and 2025	Equality monitoring	Disparity measurement	Monitors racialised workforce indicators through mandatory annual reporting.
Restoring Control over the Immigration System (UK Government 2025)	Border governance	State‐managed migration control	Frames migration within sustainability and domestic skills discourse; signals reform direction.
NHS Pastoral Care Quality Award (2022–)	Organisational integration	Employer‐led support	Standardises induction and pastoral support structures for IENs and midwives.
Stay and Thrive Programme (n.d.)	Retention strategy	Workforce stability	Supports post‐arrival integration and retention through nationally scaled guidance and resources (2024–2025). Retention framing partly driven by need to recover international recruitment investment.

*Note:* Policy documents listed refer to those included in the governance analysis. Governance framings were derived from WPR analysis of each instrument; the full six‐question analysis for documents D1–D7 is presented in Supporting Information S3, and for the NHS Long Term Workforce Plan in Table 1.

Abbreviations: DHSC = Department of Health and Social Care, IEN = internationally educated nurse.

*Strand 2: Interpretative Analysis of Lived Experience*. Findings from 17 qualitative studies were extracted into a structured analytic matrix and synthesised using the thematic synthesis approach described by Thomas and Harden (2008), employed here as an initial analytic technique within the broader CQES framework rather than as its governing epistemological orientation. Coding proceeded abductively, moving iteratively between line‐by‐line engagement with the data and theoretical interrogation through Social Identity Theory and Ontological Security, with each pass refining both the empirical codes and their theoretical interpretation without predetermining analytical categories.

Through this iterative process, the primary study framings were not accepted at face value but critically interrogated, with categories reworked and reconceptualised where analytic dialogue between strands revealed assumptions, tensions or silences not visible within individual studies. Codes were then consolidated into higher synthetic constructs reflecting mediated professional legitimacy, identity renegotiation and conditional belonging, generated through critique rather than accumulation (Supporting Information S4). The qualitative findings were read against the governance categories from Strand 1 to identify where experiential patterns confirmed, complicated or exceeded what policy representations would predict.

*Integration and Iterative Mapping*. Following completion of both strands, iterative constant comparison was used to map policy framings including operational reliance, strategic recalibration and sustainability discourse against experiential patterns such as transitional status and conditional permanence. This process produced two culminating analytical outputs: Table 3, which maps intersections between governance representations and lived professional trajectories, and Table 4, which extends analysis to the organisational strategies, structural constraints and identity implications these governance tensions generate for nursing leadership.

**Table 3:** Governance representations and their manifestation in the lived professional trajectories of internationally educated nurses.

Governance framing/discursive logic	Manifestation in lived experience (status disruption)	Identity response (adaptive reconstruction and negotiated belonging)	Empirical sources	Interpretive lens
Emphasis on competence verification, adaptation requirements and recognition of prior experience during NHS entry.	Transitional deskilling, restricted autonomy and delayed recognition of previous expertise during early transition.	Strategic compliance with regulatory and workplace expectations, followed by gradual re‐establishment of professional confidence and role legitimacy.	Stubbs (2017) Okougha and Tilki (2010), Alexis and Shillingford (2012, 2015), Pressley et al. (2023), Adhikari and Melia (2015) and Al‐Hamdan et al. (2015)	Social Identity Theory
Institutional inclusion rhetoric operating within assimilation‐oriented professional cultures.	Accent discrimination, racialisation, cultural misrecognition and perceived marginalisation within clinical and organisational settings.	Adaptive conformity to dominant norms alongside development of hybrid professional identities and selective resistance.	Batnitzky and McDowell (2011), Likupe (2015), Okougha and Tilki (2010), Alexis (2013), Spiliopoulos and Timmons (2023), Ugiagbe (2024) and Likupe (2013)	Social Identity Theory
International recruitment within shortage contexts intersects with migration as a strategy for personal, familial and professional security.	Work decisions shaped by the search for stability, family well‐being and longer term security, with employment conditions affecting settlement confidence.	Reframing migration as an economic and familial strategy prioritising stability while negotiating professional continuity and belonging.	Smith and Gillin (2021), Bidwell et al. (2014), Adhikari (2013) and Spiliopoulos and Timmons (2023)	Ontological Security
Contemporary recruitment and retention discourse foregrounds the value of IENs during periods of workforce pressure.	Ongoing challenges in integration, support and career progression despite policy emphasis on international recruitment.	Construction of resilient professional identities integrating persistence, adaptation and aspirations for longer term advancement.	Pressley et al. (2023, 2025); Spiliopoulos and Timmons (2023) and Ugiagbe (2024)	Ontological Security

*Note:* Empirical sources refer to studies included in the thematic synthesis (Supporting Information S2). Policy representations derived from WPR analysis (Tables 1 and 2). Interpretive lenses applied throughout.

**Table 4:** Governance tensions linking organisational strategies, structural constraints and identity implications for internationally educated nurses.

Organisational strategy	Structural constraint	Potential identity implication	Evidential basis	Interpretive lens
Induction and Transition (CBT/OSCE pathway)	Extended regulatory verification and employer‐supervised pre‐registration candidacy	Potential for prolonged transitional positioning and mediated professional legitimacy	Stubbs (2017), Alexis and Shillingford (2012, 2015), Al‐Hamdan et al. (2015), NMC Test of Competence and NHS LTWP (pp. 8, 15)	Social Identity Theory
Stay and Thrive/Family Integration Initiatives	Visa dependency and settlement eligibility rules	Experiences of conditional permanence and employment‐contingent security	Smith and Gillin (2021), Bidwell et al. (2014), Adhikari and Melia (2015), Health and Care Worker Visa and UK Government (2025)	Ontological Security
Mentorship and Supervision Structures	NMC Test of Competence requirements and employer‐supervised pre‐registration candidacy embedding hierarchical oversight and constrained professional authority	Potential constraints on professional voice and negotiated authority	Alexis and Shillingford (2012, 2015), Likupe (2015), Batnitzky and McDowell (2011), NHS Employers (2025) and NMC Test of Competence	Social Identity Theory
Pastoral Care and Recognition Frameworks	Resource limitations and structural workforce pressures	Recognition‐protection gap between symbolic appreciation and institutional safeguards	Pressley et al. (2023, 2025), Ugiagbe (2024), Spiliopoulos and Timmons (2023) and NHS Pastoral Care Quality Award	Ontological Security
Career Development Frameworks	Domestic expansion priorities and pipeline recalibration	Perceived restrictions in progression pathways and deferred leadership trajectories	Ugiagbe (2024), Likupe (2015), Pressley et al. (2025), NHS LTWP (pp. 7, 15) and WRES (NHS England (2024, 25)	Social Identity Theory

*Note:* Governance tensions are interpreted as structural rather than organisational. Evidential sources include both qualitative studies (Supporting Information S2) and policy instruments (Table 2).

Abbreviations: NHS LTWP = NHS Long Term Workforce Plan (NHS England 2023), WRES = Workforce Race Equality Standard (NHS England 2024, 2025).

Interpretive claims were retained only where patterns were recurrent across studies, capable of standing independently of the policy analysis strand and theoretically coherent when examined through Social Identity Theory and Ontological Security (Timmermans and Tavory 2012). Where qualitative patterns converged with, complicated or exceeded policy representations, this was treated as an analytical finding.

Data extraction was conducted using a structured analytic matrix developed to capture findings relevant to professional experience, identity negotiation, governance positioning and adaptation. The matrix was piloted on two included studies prior to full extraction to ensure consistent and complete capture of relevant material. Although extraction was led by the lead author, the completed analytic matrix was subsequently reviewed in full by an author with expertise in WPR application, who assessed the adequacy and interpretive accuracy of extracted material against the source texts. Any discrepancies were resolved through discussion and consensus among the authorial team.

### 3.6 Reflexivity and Ethical Considerations

This study adopted a reflexive approach throughout the analytic process. Interpretation was shaped through collaborative dialogue among all authors with differing professional and experiential backgrounds. The lead author is an IEN, while the two reviewing authors are UK‐trained nurses. This configuration supported experiential sensitivity to IENs' positioning while maintaining interpretive balance. Reflexive memo writing and team discussion were used to distinguish interpretation from assumption and to critically examine how positionality informed analytic decisions (Ugiagbe et al. 2025; Lincoln and Guba 1985).

Ethical attention focused on avoiding misrepresentation of policy intent, respecting the contextual integrity of the original qualitative studies and recognising the secondary and interpretive nature of the synthesis.

## 4 Findings

### 4.1 Study Characteristics

Of the studies identified through the search strategy described above, 17 met the inclusion criteria (Supporting Information S2: Table 1). All were UK‐based, published between 2010 and 2025 and focused on the experiences of IENs within the United Kingdom.

Fifteen studies employed qualitative designs, predominantly interviews and/or focus groups, while a smaller number used ethnography, phenomenology, interpretative phenomenological analysis, grounded theory or content analysis. Two studies used mixed‐methods designs, from which only the qualitative findings were extracted and synthesised.

Participants were drawn from a broad range of source countries across Africa, South and Southeast Asia, the Caribbean and the Middle East, with several studies including multinational cohorts. Sample sizes ranged from 8 to 773 participants, reflecting the inclusion of both small interview‐based studies and two large‐scale surveys.

Full study‐level details, including design, sample, country of origin, setting and thematic focus, are presented in Supporting Information S2.

### 4.2 Strand 1: Policy Discourse Analysis

Table 1 presents the WPR analysis of the NHS LTWP, interrogating the problem representations the Plan constructs. Section 4.2‐1 develops the analytical implications and Section 4.2‐2 extends the analysis across the full governance domain.

#### 4.2.1 WPR Analysis of the NHS Long Term Workforce Plan

By positioning domestic expansion as the primary route to workforce sustainability, the Plan implicitly hierarchises qualification pathways, locating domestic training as foundational and internationally acquired expertise as supplementary. This assumption is not stated explicitly; it is embedded within the logic of self‐sufficiency itself, reproduced through what the policy takes for granted rather than what it declares. Equally significant are the silences: the Plan engages extensively with numerical targets and pipeline projections while offering minimal engagement with the professional implications for IENs already embedded within the NHS. When international recruitment is framed as transitional, the symbolic positioning of IENs shifts accordingly. The policy does not construct them as unwelcome; it constructs them as necessary now but reducible in the future, not through deliberate exclusion, but through the internal logic of a sustainability narrative that takes reduced international reliance as its normative endpoint (NHS England 2023). This temporality constitutes the paradox of self‐sufficiency: not an unintended consequence of the LTWP but a structural feature of how workforce sustainability has been discursively assembled within it.

#### 4.2.2 Layered Workforce Governance

WPR analysis of the eight policy documents identified eight governance framings, each capturing the distinct function through which a policy instrument constructs IENs' positioning within the NHS workforce architecture. Table 2 organises the policy instruments according to these framings, mapping their dominant governance function and strategic framing across each layer (Full WPR Analysis Supporting Information S3).

What Table 2 makes visible is not simply a list of instruments but a governance architecture, a configuration in which eight documents, operating across different institutional sites and with different stated purposes, can be interpreted as converging to position IENs as conditionally present, procedurally differentiated and symbolically transitional within the NHS workforce. Each instrument carries its own institutional rationality. The Health and Care Worker Visa ties residency to sponsored employment. The NMC Test of Competence requires domestic verification of prior expertise. The Workforce Race Equality Standard (WRES) monitors racialised disparities without structurally addressing their governance origins. The Pastoral Care Quality Award and Stay and Thrive Programme offer organisational support downstream of the structural conditions generating the pressures they address. None of these instruments, read in isolation, appears designed to construct conditional belonging. Read together, they may be interpreted as aligning with a pattern of conditional belonging identified within the qualitative evidence.

The instruments are not coordinated by a shared intention to marginalise IENs; they are coordinated by a shared logic that positions domestic training as the normative workforce pathway and international recruitment as a managed departure from that norm. Each instrument enacts that logic differently: the Visa through residency conditionality, the NMC through competence verification, the WRES through racialised monitoring, the integration programmes through transitional support framing. What emerges is a governance environment in which IENs encounter the same fundamental positioning, necessary but provisional, valued but conditional, regardless of which institutional domain they navigate.

The WRES illustrates this most precisely. The 2023 and 2024 data analysis reports document meaningful progress in BME representation at senior levels and year‐on‐year improvements in staff confidence in career progression since 2020 (NHS England 2024, 2025). Yet shortlisting rates have worsened and the gap between overall BME clinical workforce representation and senior clinical grade representation has widened. This uneven trajectory is not a failure of measurement; it suggests that diagnostic governance may be insufficient to address structural conditions beyond its scope. The WRES monitors the outcomes of a governance architecture that the WRES itself cannot alter. This dynamic intersects with the LTWP's domestic expansion agenda: as a growing domestically trained pipeline enters at Band 5, career progression opportunities at senior clinical grades risk being shaped by a workforce planning logic oriented towards domestic pathways, which may contribute to the narrowing of progression pathways available to IENs.

The inclusion initiatives, pastoral care, retention programmes and mentorship structures, occupy a paradoxical position within this architecture. They exist within a governance context that creates immediate organisational pressures requiring management. However, they are positioned downstream of those conditions, responding to the conditions the governance environment generates, without ready access to the structural level at which those conditions are produced. An inclusion initiative may not fully address issues related to employment‐contingent permanence. A mentorship programme cannot counteract the symbolic positioning embedded in the LTWP's self‐sufficiency framing. The NHS Pastoral Care Quality Award therefore appears in Table 4 as an organisational strategy rather than a governance instrument, a distinction that reflects not a difference in importance but a difference in structural position. It responds to the tensions the governance architecture generates rather than participating in their production. Understanding this distinction is critical for nursing leadership: the governance environment does not merely constrain what organisations can do for IENs; it shapes what belonging itself means within those organisations.

### 4.3 Strand 2: Interpretative Analysis of Lived Experience

Thematic synthesis of the 17 included studies generated three synthetic constructs: mediated professional legitimacy, identity renegotiation and conditional belonging, each of which is developed interpretively in the subsections that follow. The full thematic synthesis matrix, including sub‐themes, descriptive characteristics and contributing studies, is presented in Supporting Information S4.

#### 4.3.1 Mediated Professional Legitimacy: Status Disruption and Professional Repositioning

Regulatory verification, mandatory competence assessment and temporary repositioning below prior levels of responsibility, the governance logic the NMC Test of Competence enacts at the organisational level, are formally constructed as competence assurance mechanisms. Although the formal supervised practice period of the Overseas Nurses Programme has since been replaced by the NMC Test of Competence pathway, comprising CBT, OSCE assessment and employer‐supervised pre‐registration candidacy, the underlying governance logic remains structurally unchanged: internationally acquired expertise requires domestic verification before professional legitimacy can be accorded. Status disruption is therefore not incidental to integration; it appears strongly shaped by structural conditions embedded within the regulatory architecture through which IENs enter NHS practice.

The governance logic identified in the WPR analysis finds its clearest organisational expression across the review period in accounts documenting how the entry pathway rendered prior clinical expertise invisible regardless of its specific form. Drawing on studies conducted across both the ONP era and the current Test of Competence pathway, a recurrent pattern is identified. Stubbs (2017) found that supervised practice among Indian nurses on the Overseas Nurses Programme was experienced not as professional development, but as institutional revalidation of competence already demonstrated in clinical leadership roles in their countries of origin.

This pattern was not unique to a particular national origin or entry route. Alexis and Shillingford (2012, 2015) documented near‐identical dynamics among Jamaican and Filipino neonatal nurses in London, where prior specialist expertise went unrecognised until domestically validated, producing a period of enforced professional subordination that the governance architecture required but the nurses themselves experienced as status erasure rather than integration. Nurses who had led teams and exercised autonomous clinical judgement in their countries of origin found themselves repositioned within supervised, hierarchically constrained roles, reflecting not individual deficiency but a governance configuration that significantly delays recognition of expertise acquired outside the domestic training pipeline (Alexis and Shillingford 2012, 2015; Adhikari and Melia 2015; Stubbs 2017).

The pre‐registration candidacy period therefore continues to enact the same governance logic identified in the WPR analysis. In this representation, domestically acquired verification precedes professionally accorded legitimacy, regardless of the specific pathway through which that requirement is administered (NHS Employers 2025).

#### 4.3.2 Identity Renegotiation: Adaptive Reconstruction and Hybrid Identity Formation

Adaptive strategies, including modification of communication styles, pursuit of locally recognised qualifications and selective assimilation into dominant professional cultures, are frequently framed as resilience (Alexis 2013; Pressley et al. 2023). This framing is incomplete. Read critically, these strategies reveal the asymmetry of the integration relationship: the burden of adaptation falls consistently and disproportionately on IENs rather than on the governance systems receiving them (Batnitzky and McDowell 2011; Likupe 2013, 2015).

The asymmetry of the integration relationship is documented with particular clarity by Batnitzky and McDowell (2011), whose study of Caribbean and South Asian nurses identified systematic racialisation of professional interactions alongside blocked career trajectories, dynamics that nurses navigated not by challenging the institutional environment but by developing adaptive strategies designed to demonstrate cultural competence on the organisation's own terms. Alexis (2013) describes a similar process among nurses from the Philippines, South Africa and the Caribbean, where adaptive conformity to dominant clinical norms coexisted with the development of hybrid professional identities that preserved prior expertise and cultural knowledge in a form that was neither fully visible nor fully abandoned. The burden of this reconstruction fell entirely on the nurses rather than on the institutional cultures receiving them. Adaptive reconstruction is therefore not simply a psychological process of professional integration; it may be understood as a response to a discursive environment in which the terms of professional legitimacy are defined by the dominant workforce pathway (Bacchi 2009).

#### 4.3.3 Conditional Belonging: Negotiated Belonging and Conditional Permanence

The material dimension of conditional belonging is documented most precisely by Smith and Gillin (2021), whose study of Filipino nurses found that migration to the United Kingdom was explicitly motivated by Ontological Security‐seeking, defined as the desire for professional and personal stability. Yet this aspiration was experienced within an employment and immigration environment in which residency rights were tied to sponsored employment, rendering the security sought structurally dependent on conditions outside the nurses' control.

Spiliopoulos and Timmons (2023) extend this analysis into the post‐Brexit context, documenting how nurses from both EU and non‐EU countries described themselves as tolerated rather than fully belonging: valued for present labour contribution while remaining uncertain about the permanence of their institutional place.

Together these accounts suggest that conditional belonging may be less a perception requiring correction through better communication than an interpretively grounded reading of a governance environment that is, in material and symbolic terms, experienced as conditional. IENs describe not uncertainty about whether they are valued, many accounts confirm they are, but uncertainty about whether that value will persist and, on whose terms (Spiliopoulos and Timmons 2023; Bidwell et al. 2014).

#### 4.3.4 Synthesis Across the Three Constructs

These constructs were not linear or universally experienced. Their intensity and persistence varied according to organisational culture, trust‐level governance, employment conditions and migration context. While some nurses achieved sustained professional stability and Ontological Security, particularly where recognition and progression opportunities were available (Al‐Hamdan et al. 2015; Smith and Gillin 2021; Pressley et al. 2023), the recurrence of similar identity patterns across 15 years of qualitative evidence, diverse national origins and varied clinical settings warrants a structural rather than incidental analytical account. Individual variation in outcome does not dissolve the pattern; it confirms that where conditions of recognition, progression and stability were present, nurses were able to achieve what the governance architecture otherwise makes difficult to achieve.

Social Identity Theory accounts for why these patterns recur at the group level: when governance discourse consistently positions internationally educated pathways as supplementary rather than foundational, it may provide a context within which mediated legitimacy, adaptive conformity and negotiated belonging become intelligible across diverse organisational contexts (Tajfel and Turner 1979). Ontological Security accounts for why these patterns persist temporally: the governance environment may progressively narrow the conditions of predictability upon which stable professional identity depends, meaning that the disruption is not resolved through time and familiarity but reproduced through the structural conditions within which professional life continues to be lived (Giddens 1991).

Together the three dynamics documented in this strand, mediated professional legitimacy, identity renegotiation and conditional belonging, are not separate phenomena but interconnected expressions of a single governance logic that constructs IENs as conditionally present within a workforce that structurally depends on their permanence. The governance configuration examined in Section 4.2 does not merely coexist with these experiential patterns; the two appear consistent with one another and provide one possible interpretive context through which they may be understood, a connection developed further in the integration analysis that follows.

### 4.4 Integration: Policy Narratives and Lived Experience in Dialogue

This section brings both strands into direct analytical dialogue, reading the terrain where governance framing and lived professional trajectories intersect, diverge and mutually constitute one another not as description but as an interpretive account of how policy discourse is interpreted as shaping the conditions within which the identity dynamics documented in Section 4.3 are structurally reproduced.

The qualitative evidence suggests that status disruption is not incidental to NHS entry but a recurrent outcome of a regulatory architecture that appears to delay recognition of internationally acquired expertise. It complicates governance representations by indicating that adaptive identity reconstruction, often framed within policy discourse as evidence of successful integration, may be better understood as a response to asymmetric institutional conditions rather than an indicator of genuine professional belonging. It also raises questions that governance representations do not anticipate. Across the included studies, conditional belonging does not appear to be a transitional experience that resolves through time or organisational support. Instead, it emerges as a persistent pattern within a governance environment that has not been configured to accommodate the permanence on which it nevertheless relies.

### 4.5 Governance Tensions and the Paradox of Self‐Sufficiency

Table 4 represents the culminating analytical move, asking what the governance configuration identified across the preceding sections demands of nursing leadership and what it makes structurally difficult to deliver.

The tensions mapped in Table 4 are not organisational failures; they are consistent with a multilevel governance architecture oriented towards partially incompatible objectives. Induction programmes operate within regulatory requirements that extend the transitional period. Mentorship structures operate within hierarchical clinical environments that may constrain professional voice. Career development frameworks operate within a national planning environment actively recalibrating its domestic pipeline. The recognition gap between symbolic appreciation and institutional safeguarding reflects the structural position of integration initiatives downstream of the conditions generating the pressures they address.

These tensions fall unevenly. The weight of navigating them is borne disproportionately by IENs whose professional identity and sense of permanence are continuously shaped by governance signals they cannot alter. For nursing leadership, the governance environment appears to create a structurally ambiguous position: nurse managers are simultaneously agents of organisational inclusion and representatives of a governance system that conditions the very belonging they are tasked with supporting. Leadership practices that recognise IENs as enduring contributors and advocate for equitable progression are meaningful, but their capacity to moderate structural conditions depends on whether they are exercised within policy frameworks genuinely oriented towards unconditional inclusion. Rather than being resolved at the organisational level, the paradox appears to be mediated through organisational structures.

## 5 Discussion

### 5.1 Workforce Self‐Sufficiency and Discursive Positioning

The paradox of self‐sufficiency functions as discursive governance: the LTWP does not merely administer workforce supply but can be understood as constructing a representation of who IENs are within the projected NHS workforce, what problem their presence represents and what kind of future their continued participation implies. By framing sustainability as dependent on reducing international recruitment, the Plan appears to position domestic qualification as the normative pathway to workforce membership, with international recruitment constructed as a departure from that norm.

This hierarchisation is not stated explicitly; it appears embedded within the logic of self‐sufficiency itself, which tends to take for granted that reduced international reliance is inherently more sustainable, more ethical and more resilient. The WPR analysis makes this assumption visible; the question is what it means when it circulates as governance discourse within an NHS that has depended on IENs for generations.

It bears acknowledging that the self‐sufficiency agenda carries legitimate rationale: reducing dependence on international recruitment addresses genuine ethical concerns about source country workforce depletion (World Health Organization 2010), builds resilience against global labour market volatility and responds to the unsustainable marginal costs of sustained international recruitment at scale (NHS England 2023).

Acknowledging this rationale does not, however, alter what the self‐sufficiency framing may do discursively within the governance environment it enters. The symbolic positioning of IENs as transitional does not appear to land in a neutral institutional environment. Rather, it intersects with organisations where differential recognition, constrained autonomy and hierarchical positioning are consistently documented across 15 years of qualitative evidence.

Social Identity Theory illuminates this intersection (Tajfel and Turner 1979): macro‐level policy discourse may reinforce the distinction between domestic and internationally educated pathways, potentially reinforcing existing forms of status differentiation. Post‐Brexit scholarship deepens this analysis: the paradox of self‐sufficiency may not introduce conditional belonging into a previously secure environment so much as reinforce a conditionality that the evidence suggests was already present (Spiliopoulos and Timmons 2023).

For nursing leaders, the discursive signals embedded within self‐sufficiency governance may not be fully addressable through organisational communication alone. They appear to reflect conditions requiring active interrogation and counterbalancing at governance levels beyond the organisation: within integrated care systems, professional regulatory bodies and national workforce policy processes. This is a significantly more demanding form of leadership than the LTWP's own framing implies (Edmondson 2018).

### 5.2 Identity, Conditional Belonging and Professional Continuity

The intersection between policy discourse and lived experience is most analytically consequential at the theme of conditional permanence. Visa sponsorship dependency, employment‐contingent settlement rights and gaps between symbolic valorisation and material support produce a form of professional life in which belonging must be continuously earned. This reflects the material realities of the governance environment: the Health and Care Worker Visa ties residency to sponsored employment (UK Government 2020); the LTWP signals progressive reduction of structural reliance on international recruitment. Together, these instruments may contribute to a governance environment in which present indispensability does not translate into future security.

The qualitative evidence documents a pattern sufficiently consistent across studies, settings and national origins to warrant interpretation beyond individual circumstance: IENs repeatedly describe professional life as conditional, contingent on regulatory validation, employer goodwill, immigration status and institutional recognition that appears to require continuous renewal. Social Identity Theory explains the group‐level dimension (Tajfel and Turner 1979): when governance discourse positions internationally educated pathways as supplementary rather than foundational, it may reinforce conditions associated with the status differentiation documented across the evidence base. Resilience and adaptive reconstruction, frequently celebrated as evidence of agency (Alexis 2013; Pressley et al. 2023), may be more fully understood as responses to asymmetric institutional conditions.

Ontological Security extends this analysis where Social Identity Theory cannot reach. Giddens (1991) argues that Ontological Security depends on a sufficiently predictable social environment. The governance configuration, through visa conditionality, extended regulatory verification, self‐sufficiency signalling and recalibrated career pathways, may be experienced as narrowing the conditions for that predictability. Smith and Gillin (2021) document Filipino nurse migration as explicitly Ontological Security‐seeking; when those aspirations encounter an environment in which residency ties to sponsored employment and national strategy signals reduced reliance, the result appears to be a sustained challenge to the continuity of professional self. The 2025 Immigration White Paper's proposals to extend qualifying periods for indefinite leave to remain may intensify rather than resolve this challenge (UK Government 2025).

The recurrent pattern of conditional belonging across the qualitative literature may be less a subjective perception than an interpretively grounded reading of a governance environment that is, in material and symbolic terms, experienced as conditional (Spiliopoulos and Timmons 2023; Ugiagbe 2024).

IENs are not uncertain about being valued; the evidence is consistent on that point. What remains uncertain is whether that value endures, and who controls the terms. Addressing this may require not only better messaging about IEN value but critical engagement with the governance instruments that appear to shape the conditions within which belonging is experienced as conditional in the first place. When the governance horizon within which IENs must plan their professional futures remains uncertain, the capacity to maintain a coherent and continuous professional self may be less a matter of individual resilience than of the governance conditions within which professional life is structured.

### 5.3 Multilevel Governance and Organisational Mediation

The governance configuration is organised around a tension: organisations are asked to integrate and retain IENs now, while national policy projects a future in which that integration investment may carry diminishing structural priority. Organisational integration initiatives, including induction, pastoral care, mentorship and retention schemes, represent genuine institutional commitments to supporting IENs within the NHS. However, they sit downstream of the governance conditions generating the pressures they address, and their capacity to reach the structural level at which those conditions operate is limited. An inclusion initiative may have limited purchase on employment‐contingent permanence; a mentorship programme may struggle to counteract the symbolic positioning of IENs as transitional within a national workforce strategy. The conditionality embedded in immigration frameworks, regulatory verification requirements and national workforce recalibration operates at a level that organisational programmes are not readily configured to alter (Lanada and Culligan 2024; Rajpoot et al. 2024).

Social Identity Theory illuminates why (Tajfel and Turner 1979): macro‐level discourse constructing international recruitment as transitional is unlikely to be neutralised by organisational inclusion rhetoric alone. Ontological Security explains what the temporal disjunction may produce at the level of professional self (Giddens 1991): IENs are asked to invest in NHS careers within an environment in which their long‐term place is subject to projections they have limited capacity to influence and conditions that remain largely outside their control.

For nursing leaders, the governance environment generates not merely practical complexity but ethical complexity that practice alone cannot fully resolve. Meaningful commitment to IENs' belonging may therefore require engagement beyond the organisational level, including advocacy within integrated care systems and national policy processes for governance frameworks oriented towards more genuinely unconditional inclusion.

### 5.4 Theoretical and Conceptual Contributions

This study advances nursing management scholarship in three ways. First, the paradox of self‐sufficiency offers a context‐specific conceptual contribution. While tensions between continuing reliance on IENs and policy ambitions for greater workforce self‐sufficiency have been documented within the broader global nursing migration literature (Buchan et al. 2022; Hynes et al. 2025; Kingma 2006), their theorisation within the specific policy context of the first comprehensive 15‐year NHS workforce strategy represents a distinct analytical contribution not yet evident in the literature.

Second, integrating Bacchi's WPR framework with Social Identity Theory and Ontological Security constitutes a methodological contribution: this combination has not been widely applied to examine workforce policy as discursive governance with identity effects across multiple institutional levels, and its application here offers a transferable analytical approach for nursing workforce scholarship more broadly (Bacchi 2009; Tajfel and Turner 1979; Giddens 1991).

Third, the multilevel governance analysis situates the LTWP within its interconnected socio‐political, immigration, regulatory and organisational environment, bringing domains that have tended to be examined separately into systematic analytical dialogue and offering nursing leaders a more comprehensive account of the structural conditions within which their practices operate.

### 5.5 Implications for Nursing Management

These findings carry important implications for nursing leadership, situated within recognition that nurse managers operate within the same multilevel governance constraints this study has analysed.

Immediate organisational actions centre on recognition and integration practice: explicitly acknowledging the prior expertise IENs bring during clinical placement and appraisal processes; extending pastoral support beyond initial arrival into the second and third year of employment (Pressley et al. 2023, 2025), and ensuring psychological safety frameworks actively challenge racialised workplace dynamics rather than simply promoting generic inclusion (Edmondson 2018).

Medium‐term system‐level actions require advocacy beyond the individual organisation: nurse leaders engaging integrated care system workforce planning discussions to make visible the retention consequences of self‐sufficiency signalling, and Trust boards commissioning WRES‐informed action plans (NHS England 2025) that distinguish between BME representation targets and IEN‐specific retention and progression outcomes.

Long‐term policy‐level actions require sustained engagement with national governance: nursing professional bodies advocating for immigration policy stability for sponsored NHS workers (UK Government 2020, 2025), and workforce planning frameworks that articulate IEN contributions as structurally enduring rather than transitionally necessary.

### 5.6 Limitations and Future Directions

This review synthesised published qualitative research and policy documents without generating primary data. Findings are interpretive and theoretically informed; alternative readings remain possible. The search strategy was limited to four peer‐reviewed databases. Grey literature was not searched through database aggregators; targeted organisational searches were conducted across The King's Fund, Nuffield Trust and The Health Foundation, and did not yield evidence that would materially alter the analytical corpus. The selected documents were confirmed as sufficient to represent the relevant governance landscape through the conceptual sufficiency criterion described in Section 3.2.

Full‐text screening was conducted by the lead author following almost perfect agreement at title and abstract stage, with all decisions verified by a second independent reviewer. The authorial team's prior collaborative experience in meta‐ethnographic synthesis provided additional interpretive grounding, and evidence suggests that single screening miss rates are substantially lower among experienced reviewers where verification is applied (Waffenschmidt et al. 2019). Dual extraction as standard practice would nonetheless further strengthen replicability in future syntheses.

Future research should examine longitudinal career trajectories of IENs during domestic workforce expansion, evolving interpretations of permanence and belonging as the LTWP is implemented, and how leadership practices mediate tensions between workforce recalibration strategies and organisational reliance on IENs.

## 6 Conclusion

This study has examined how NHS workforce governance narratives intersect with the professional identity trajectories of IENs, producing not a description of experiences nor an evaluation of policy, but a critical account of how governance discourse and professional identity are mutually implicated within a specific governance configuration.

The paradox of self‐sufficiency, the coexistence of operational indispensability and strategic transitional positioning, is the organising concept through which that configuration has been examined. It does not claim the LTWP is designed to exclude IENs, nor that conditional belonging is a deliberate governance outcome. What it does claim is that the structural coexistence of present reliance and projected future reduction generates a governance environment in which conditional belonging may be understood as associated with structural conditions identified across the governance landscape, alongside differential recognition, constrained autonomy and hierarchical positioning documented consistently across 15 years of qualitative evidence. Viewed through Social Identity Theory, this configuration constructs group positioning effects that organisational inclusion rhetoric alone cannot neutralise. Viewed through Ontological Security, the governance environment may be experienced as reducing the stability and predictability upon which professional identity is often built. Integrating these frameworks with Bacchi's WPR approach enables the study to move beyond documenting what IENs experience towards examining how governance discourse shapes the institutional conditions within which those experiences are reproduced.

If conditional belonging is strongly shaped by structural conditions rather than individually or organisationally generated, then addressing it requires engagement at the level of governance design rather than solely organisational practice. The paradox of self‐sufficiency offers a conceptual framework applicable beyond the NHS context; the theoretical integration demonstrated here equips nursing workforce scholarship to examine policy as discursive governance with identity effects across institutional levels, and the synthesis suggests that the patterns documented in the literature are closely associated with broader structural conditions and may continue to recur if those conditions remain substantially unchanged.

Workforce sustainability and professional belonging are not competing priorities; they are mutually dependent conditions for a health system that continues to rely, and will for the foreseeable future continue to rely, on the expertise of nurses educated beyond its borders. The next iteration of NHS workforce planning, whether a revision of the LTWP or its successor, presents a specific opportunity to operationalise this recognition: to reframe IENs not as a transitional workforce dependency to be reduced but as a structurally permanent feature of a health system that has always been, and will continue to be, internationally constituted.

## Author Contributions

J.C.N. conducted the literature search, review, data extraction and primary analysis. O.B. contributed to title and abstract screening, conflict resolution, interpretation of findings and critical revision of the manuscript. K.H. contributed to article review, interpretation of findings and critical revision of the manuscript. All authors reviewed and approved the final manuscript.

## Funding

The authors have nothing to report.

## Ethics Statement

Ethical approval was not required for this study as the research involved the analysis of publicly available policy documents and previously published studies. The study adhered to institutional research governance standards for secondary research.

## Conflicts of Interest

The authors declare no conflicts of interest.

## Use of Artificial Intelligence Tools

Rayyan.ai was used to support title and abstract screening during the review process. Microsoft Copilot was used to assist with interrogation of policy documents and for editing and organisational tasks during manuscript preparation. All screening decisions, policy interpretations, analytical judgements and final manuscript content were made and verified by the authors.

## Supporting information


Supporting File 1

Supporting File 2

Supporting File 3

Supporting File 4

## Data Availability

No new datasets were generated or analysed in this study. All sources analysed are publicly available in the published literature.
