# Supplementary material for: The Paradox of Workforce Self‐Sufficiency: Policy Narratives, Professional Identity and Internationally Educated Nurses in the United Kingdom
Source: Nurs Inq. 2026 Sep 8;33(4):e70164. doi: 10.1111/nin.70164 (PMC13554827; doi:10.1111/nin.70164)
Supplement: Supplementary file 1 [file NIN-33-e70164-s001.docx]

**Supplementary File 3**

**Full ‘What’s the Problem Represented to Be?’ Analysis of Policy and Regulatory Documents**

**Shaping Internationally Educated Nurses’ Positioning Within NHS Workforce Governance**

*Bacchi (2009) WPR Framework | Eight-Document Governance Corpus*

**Methodological Note**

This supplementary file presents the full ‘What’s the Problem Represented to Be?’ (WPR) analysis (Bacchi, 2009) for seven of the eight policy and regulatory documents constituting the governance corpus examined in this study. The eighth document—the NHS Long Term Workforce Plan (NHS England, 2023) is analysed in Table 1 of the main manuscript and is not reproduced here to avoid duplication.

Each document was read individually and interrogated through Bacchi’s six analytical questions, applied flexibly with analytical emphasis weighted according to the salience of particular representations within individual documents. Governance framings were compared across all eight documents to identify recurring problem representations and were consolidated into eight analytical categories reflecting the distinct governance functions through which internationally educated nurses’ positioning is constructed within the NHS workforce architecture, as presented in Table 2 of the main manuscript.

Each document entry below comprises: (1) a two-sentence contextualising statement identifying the document’s governance function and overall WPR interpretive claim; (2) a structured six-question WPR analysis table; and (3) an analytical note. Documents are numbered D1–D7 corresponding to their order in Table 2 of the main manuscript. The NHS Long Term Workforce Plan (Table 2, Row 1) is designated D0 and analysed separately in the main manuscript.

**D1. Code of Practice for the International Recruitment of Health and Social Care Personnel (DHSC, 2023; Updated 2025)**

***Governance Layer: Ethical Recruitment Regulation | Table 2, Row 2***

The Code of Practice governs how NHS and social care organisations recruit internationally, establishing red and amber country classifications, recruiter compliance requirements, and organisational accountability mechanisms. Read through the WPR framework, the Code constructs international recruitment as an ethically manageable practice requiring regulation rather than elimination, while its compliance architecture simultaneously normalises the transactional character of international labour sourcing and positions internationally educated nurses as subjects of ethical protection rather than professional contributors.

| **WPR Question** | **Discursive Representation** | **Indicative Evidence** | **Analytical Implications for IEN Professional Identity** |
| --- | --- | --- | --- |
| **Q1: What is the problem represented to be?** | International recruitment is positioned as ethically risky if unmanaged, requiring regulatory governance to prevent exploitation of source country health systems. | Red/amber country classification system; recruiter registration requirements; organisational accountability for compliance. | Regulatory Legitimacy: IENs are positioned as subjects of ethical protection rather than professional contributors, framing their recruitment as a managed risk rather than a valued workforce strategy. |
| **Q2: What assumptions underlie this representation?** | Global nurse migration is assumed to require external governance to be ethically defensible; source country depletion is taken as an inherent risk of international recruitment. | WHO Global Code of Practice referenced as foundational; country-of-origin classifications determine recruiter permissions. | Supplementary Positioning: The compliance framework implicitly constructs internationally educated nurses as arriving from systems requiring protection, potentially reinforcing perceptions of differential status. |
| **Q3: How has this representation come about?** | Sustained international criticism of NHS recruitment practices following high-profile sourcing from low-income countries; WHO Global Code of Practice (2010) as normative framework. | References to WHO (2010) Code; post-pandemic recruitment surge and associated ethical concerns. | Conditional Legitimacy: IENs recruited within Code compliance are ethically sanctioned; those outside compliance risk being positioned as problematic sourcing, which may produce differential symbolic status. |
| **Q4: What is left unproblematised or silent?** | The Code focuses on recruitment ethics rather than post-arrival professional recognition, career progression, or the long-term belonging of IENs once employed. | No provisions addressing IEN career development, seniority recognition, or organisational integration beyond initial employment. | Post-Arrival Silence: By focusing exclusively on sourcing ethics, the Code renders invisible the professional identity implications for nurses after recruitment. |
| **Q5: What discursive effects are produced?** | International recruitment is constructed as permissible but bounded; IENs are positioned as beneficiaries of ethical protection rather than agents of professional migration. | Compliance language frames employers as gatekeepers and IENs as subjects of governance rather than workforce members exercising professional agency. | Agency Constraint: The ethical protection discourse may reinforce perceptions of IENs as passive subjects of policy rather than active contributors, affecting professional self-positioning. |
| **Q6: How could the problem be represented differently?** | International recruitment framed as a mutual professional partnership between NHS organisations and globally mobile nursing professionals, with governance focused on equitable conditions rather than sourcing compliance alone. | Current framing centres on country-of-origin risk; an alternative would centre on bilateral professional recognition and equitable employment conditions. | Such reframing would position IENs as professional partners within a globally interconnected workforce, potentially strengthening ontological security and professional belonging. |

***D2. Health and Care Worker Visa (UK Government, 2020–)***

***Governance Layer: Immigration Governance | Table 2, Row 3***

The Health and Care Worker Visa governs the immigration pathway for internationally educated nurses entering NHS employment, linking the right to reside in the United Kingdom to sponsored employment in eligible health and care roles. Read through the WPR framework, the Visa regime constructs international labour migration as a conditional and employer-mediated privilege rather than a professional entitlement, with significant implications for the experience of conditional permanence documented in the qualitative synthesis.

| **WPR Question** | **Discursive Representation** | **Indicative Evidence** | **Analytical Implications for IEN Professional Identity** |
| --- | --- | --- | --- |
| **Q1: What is the problem represented to be?** | Migration for health and care work is positioned as a privilege requiring employer sponsorship and state sanction, framed within managed migration policy as a controlled inflow of skilled labour. | Sponsorship requirement; occupation eligibility lists; salary thresholds; employer licence conditions. | Employment-Contingent Belonging: The visa architecture constructs IENs’ right to remain as dependent on continued sponsored employment, a structure consistent with the experience of conditional permanence documented in the qualitative evidence. |
| **Q2: What assumptions underlie this representation?** | International labour migration is assumed to require state control to serve national interests; the nurse is positioned as a skilled migrant worker rather than a professional with portable credentials and independent standing. | Occupation-based eligibility; employer-controlled sponsorship; salary floor requirements linking value to wage level. | Instrumentalisation of Professional Identity: The visa’s construction of the nurse as a sponsored migrant worker may subordinate professional identity to employment status, reinforcing conditional rather than permanent belonging. |
| **Q3: How has this representation come about?** | Post-Brexit immigration policy reform replacing free movement with a points-based system; health and care designated as a shortage occupation requiring managed inflow. | Introduced following EU exit; shortage occupation classification for nursing; aligned with Tier 2 replacement framework. | Post-Brexit Conditionality: The visa regime appears to have intensified conditionality for EU-origin nurses previously covered by free movement, extending employment-contingent permanence to a broader population of internationally educated NHS nurses. |
| **Q4: What is left unproblematised or silent?** | The visa regime does not address professional recognition, career progression, or the long-term settlement aspirations of internationally educated nurses beyond employment continuation. | No provisions for professional seniority recognition; settlement pathway linked solely to employment duration rather than professional contribution. | Settlement Silence: The absence of provisions connecting professional contribution to settlement security reinforces belonging as contingent on continued employment rather than professional integration. |
| **Q5: What discursive effects are produced?** | IENs are constructed as conditional residents whose presence is sanctioned for the duration of sponsored employment; departure from sponsorship ruptures the right to remain. | Visa tied to employer; change of employer requires sponsor transfer; loss of employment creates immigration vulnerability. | Structural Precarity: The employer-contingent visa structure constructs a form of professional life in which organisational belonging and legal residence are mutually dependent, a configuration consistent with the conditional permanence documented across the qualitative evidence. |
| **Q6: How could the problem be represented differently?** | Migration for public health service work can be reframed as a professional entitlement arising from sustained NHS contribution rather than a conditional privilege dependent on employer sponsorship. This reframing relocates settlement entitlement from state‑mediated employment dependency to professional registration and public service contribution, constructing IENs as professional contributors with a public service claim to residence. | Current framing: employer-mediated conditional residence requiring continuous renewal. Alternative: professional registration-linked settlement entitlement recognising public service contribution as the basis for secure residence. | Such reframing would structurally address the employment-contingent belonging documented across the qualitative synthesis by decoupling residency security from employer-mediated sponsorship, reducing the conditional permanence associated with challenges to ontological security and long-term professional identity formation. |

**D3. NMC Test of Competence (CBT/OSCE) and Standards for Overseas Registration (NMC, 2021–, Updated)**

***Governance Layer: Professional Regulation | Table 2, Row 4***

The NMC Test of Competence governs the professional registration pathway for internationally educated nurses seeking to practise in the United Kingdom, comprising a computer-based test (CBT), an objective structured clinical examination (OSCE), and an employer-supervised pre-registration candidacy period. Read through the WPR framework, the registration pathway constructs internationally acquired nursing expertise as requiring domestic verification before legitimacy can be accorded, with direct implications for status disruption and the mediation of professional legitimacy documented across the qualitative synthesis.

| **WPR Question** | **Discursive Representation** | **Indicative Evidence** | **Analytical Implications for IEN Professional Identity** |
| --- | --- | --- | --- |
| **Q1: What is the problem represented to be?** | International nursing qualifications are positioned as requiring domestic verification to assure public protection; prior expertise is not recognised as sufficient for autonomous NHS practice without UK-specific assessment. | CBT/OSCE requirement for all overseas applicants regardless of prior seniority or specialist experience; mandatory employer-supervised pre-registration candidacy period (NHS Employers, 2025). | Credential Deficit Construction: The assessment architecture constructs internationally acquired expertise as presumptively deficient until domestically verified, a construction consistent with the status disruption documented across the qualitative synthesis. |
| **Q2: What assumptions underlie this representation?** | UK nursing standards are assumed to constitute the normative benchmark against which international competence must be measured; domestic qualification is implicitly constructed as the referent standard. | Assessment criteria aligned to UK NMC standards; no differential pathway for senior or specialist internationally educated nurses. | Domestic Standard as Norm: The assessment framework embeds the assumption that UK training constitutes the appropriate standard for NHS practice, symbolically positioning internationally acquired expertise as supplementary until verified against this norm. |
| **Q3: How has this representation come about?** | Public protection mandate of the NMC; historical concerns about variable international qualification standards; post-Bristol Inquiry professional regulation reform. | NMC statutory remit; international variation in nursing education standards; regulatory harmonisation challenges post-EU exit. | Regulatory Legitimacy: The assessment architecture carries professional legitimacy derived from the NMC’s statutory public protection mandate, making it difficult to challenge without appearing to compromise patient safety. |
| **Q4: What is left unproblematised or silent?** | The pathway does not differentiate between senior internationally educated nurses with substantial specialist experience and newly qualified nurses; all are subject to the same assessment and pre-registration candidacy requirements. | No seniority-adjusted pathway; Band 5 entry regardless of prior Band 7+ experience; pre-registration candidacy applies universally regardless of prior clinical leadership (NHS Employers, 2025). | Seniority Erasure: The absence of a differentiated pathway for experienced internationally educated nurses corresponds to the enforced professional subordination documented in the qualitative evidence, where clinical leaders were repositioned as supervised practitioners. |
| **Q5: What discursive effects are produced?** | The assessment pathway positions internationally educated nurses as temporarily incompetent practitioners whose legitimacy is contingent on successful domestic verification, producing a transitional period of reduced professional authority. | Pre-registration candidacy requirements; restricted clinical autonomy during assessment period; institutional hierarchy reinforced through supervisory relationships. | Mediated Legitimacy: The assessment architecture institutionalises the mediation of professional legitimacy, requiring internationally educated nurses to demonstrate competence already exercised in their countries of origin before organisational recognition is accorded. |
| **Q6: How could the problem be represented differently?** | International nursing registration framed as a process of professional equivalence recognition rather than competence verification, with differentiated pathways for experienced practitioners and senior clinical leaders. | Current framing: universal competence verification regardless of experience. Alternative: experience-adjusted recognition pathway acknowledging prior specialist contribution. | Such reframing would reduce the enforced professional subordination produced by the current pathway, supporting more rapid re-establishment of professional identity and reducing status disruption that delays full integration. |

**D4. Workforce Race Equality Standard (NHS England, 2024, 2025; 2023 and 2024 Data)**

***Governance Layer: Equality Monitoring | Table 2, Row 5***

The Workforce Race Equality Standard (WRES) requires NHS organisations to collect and report annually on nine indicators of racial equality in workforce experience and representation, with the 2023 and 2024 data analysis reports constituting the eighth and ninth publications since mandation. Read through the WPR framework, the WRES constructs racial inequality as a measurable and governable organisational performance problem, positioning diagnostic transparency as the primary governance response while locating accountability at trust level rather than within the national policy architecture generating the disparities it records.

| WPR Question | Discursive Representation | Indicative Evidence | Analytical Implications for IEN Professional Identity |
| --- | --- | --- | --- |
| Q1: What is the problem represented to be? | Racial inequality in NHS workforce experience and representation is constructed as an organisational measurement problem requiring annual data collection and reporting to drive improvement. | Nine mandatory indicators covering representation, progression, disciplinary processes and staff experience; annual trust-level submission via the Data Collection Framework with a 100% return rate achieved; four indicators drawn from workforce data, four from the NHS Staff Survey, one from board composition. | Measurement as Governance: By framing racial inequality as a measurement problem, the WRES positions data collection as the primary governance response, which may substitute diagnostic activity for structural change. |
| Q2: What assumptions underlie this representation? | Racial inequality is assumed correctable through organisational measurement and transparency; reporting is assumed to generate accountability sufficient to drive behavioural change. | Sustained measurement coincided with deterioration on structural indicators: trusts at which white applicants were significantly more likely to be appointed from shortlisting rose from 71% to 76% to 80%; disciplinary disparity rose from 46% to 51% of trusts; the national likelihood ratio for appointment from interview has remained broadly unchanged since 2016. | Accountability Assumption: The persistence of gatekeeping disparities across successive reporting cycles suggests that measurement and transparency may not, on their own, generate sufficient organisational leverage over the conditions producing differential progression. |
| Q3: How has this representation come about? | NHS equality and diversity agenda; documented BME leadership underrepresentation; post-Windrush attention to racial injustice in public institutions; subsequent alignment with national workforce strategy. | Introduced 2015 and refined through annual iterations; integrated with the EDI improvement plan; the 2023 report positions its data as a snapshot of progress against ambitions set out in the NHS Long Term Workforce Plan. | Convergent Framing: The WRES's self-positioning against LTWP ambitions locates equality monitoring within the same workforce strategy whose self-sufficiency logic this analysis identifies as shaping IEN positioning, aligning the monitoring instrument with the governance architecture it measures. |
| Q4: What is left unproblematised or silent? | Disaggregation of internationally educated staff is treated as discretionary rather than as a governance requirement: reported in 2023, discontinued in 2024, with disparity otherwise monitored through an aggregate BME category. | The 2023 report includes a dedicated section on internationally recruited nurses, midwives and nursing assistants, drawing on a staff survey question introduced in November 2021, and finds that BME nurses and nursing assistants — whether domestically or internationally recruited — alongside internationally recruited white staff, report poorer NHS working experiences. No equivalent section appears in the 2024 report; no indicator requires IEN disaggregation. | Discontinued Visibility: The 2023 disaggregation demonstrates that IEN-specific monitoring is feasible within existing data collection, which suggests that its absence from the 2024 report reflects the discretionary status of that disaggregation rather than a technical constraint. IEN-specific progression barriers thereby remain outside the governance framework's routine field of vision. |
| Q5: What discursive effects are produced? | Racial inequality is constructed as a trust-level organisational performance issue rather than a systemic governance outcome, positioning NHS organisations as primarily responsible for disparities generated by national policy and regulatory architecture. | Trust-level reporting, organisational action plans and board-level accountability without equivalent national governance accountability; staff-experience indicators improved over the period while workforce-data indicators governing recruitment and progression deteriorated. | Organisational Responsibility: The divergence between improving experience measures and deteriorating structural measures is consistent with an accountability architecture directed downward to organisations, which may leave the conditions generating differential progression outside the reach of the instrument recording them. |
| Q6: How could the problem be represented differently? | Racial inequality in the NHS could be reframed as a structural governance outcome rather than an organisational performance deficit, locating disparity within immigration, regulatory and workforce planning architectures and treating IEN disaggregation as a standing requirement. | Current framing: racial inequality as an organisational measurement and transparency problem, with BME aggregation and discretionary IEN reporting. Alternative: racial inequality as a structural governance outcome requiring national policy accountability and sustained IEN-specific indicators, the feasibility of which the 2023 reporting cycle already demonstrates. | Such reframing would redirect governance attention from organisational compliance toward the structural conditions associated with racial disparity, and would render IEN-specific progression barriers continuously visible rather than dependent on discretionary inclusion within a given reporting cycle. |

**D5. Restoring Control over the Immigration System (UK Government, 2025)**

***Governance Layer: Border Governance | Table 2, Row 6***

The 2025 Immigration White Paper sets out proposed reforms to the immigration system, including proposals to extend the qualifying period for indefinite leave to remain, tighten salary thresholds for skilled worker visas, and reduce net migration. Read through the WPR framework, the White Paper constructs migration as a problem of national control requiring correction, creating a convergent governance signal that reinforces the LTWP’s self-sufficiency narrative and compounds the conditional permanence experienced by internationally educated NHS nurses.

| **WPR Question** | **Discursive Representation** | **Indicative Evidence** | **Analytical Implications for IEN Professional Identity** |
| --- | --- | --- | --- |
| **Q1: What is the problem represented to be?** | Immigration is positioned as having been insufficiently controlled, with net migration levels constructed as unsustainably high and requiring reduction through tighter eligibility criteria and extended settlement qualifying periods. | Proposals to extend ILR qualifying period from five to ten years; salary threshold increases; points-based system tightening. | Extended Conditionality: If implemented, the proposed extension of the ILR qualifying period would defer settlement security by an additional five years beyond current policy, which may intensify the conditional permanence experienced by internationally educated nurses. |
| **Q2: What assumptions underlie this representation?** | Net migration reduction is assumed to be inherently beneficial for national resilience and public service sustainability; domestic workforce development is positioned as the preferred alternative to international recruitment. | References to domestic skills development; alignment with LTWP self-sufficiency narrative; framing of migration reduction as system restoration. | Convergent Governance Logic: The White Paper’s domestic-first framing reinforces the LTWP’s self-sufficiency narrative, creating a convergent governance signal that internationally educated nurses’ long-term presence is transitional rather than permanent. |
| **Q3: How has this representation come about?** | Post-Brexit political environment; public concern about net migration levels; alignment between Home Office border control objectives and NHS England workforce self-sufficiency ambitions. | 2023 net migration statistics; Conservative and Labour party convergence on migration reduction; LTWP domestic expansion as parallel policy signal. | Policy Convergence: The alignment between immigration reduction and workforce self-sufficiency may create a mutually reinforcing governance environment in which internationally educated nurses receive convergent signals of transitional rather than permanent status from both immigration and workforce policy simultaneously. |
| **Q4: What is left unproblematised or silent?** | The White Paper does not address the implications of extended ILR qualifying periods for NHS workforce stability, staff retention, or the professional belonging of internationally educated nurses currently employed in the NHS. | No NHS workforce impact assessment included; no engagement with retention implications of extended settlement uncertainty for currently employed IENs. | Workforce Silence: The absence of workforce impact analysis reproduces the governance silence identified in the LTWP, where policy decisions with significant implications for internationally educated nurses’ professional lives are made without engagement with those implications. |
| **Q5: What discursive effects are produced?** | Internationally educated NHS workers are positioned within a migration management discourse that constructs their continued presence as subject to ongoing state sanction rather than professional entitlement, reinforcing employment-contingent belonging. | Proposed ILR extension; salary threshold increases affecting some nursing grades; framing of health and care workers within broader migration reduction agenda. | Compounded Precarity: The White Paper’s proposals may compound the structural precarity associated with the visa regime, extending the period during which internationally educated nurses must maintain continuous sponsored employment to access settlement security. |
| **Q6: How could the problem be represented differently?** | Migration for public service work can be reframed as a governance resource rather than a control problem. This perspective challenges net‑migration reduction logic by construing internationally educated nurses’ sustained NHS contribution as a governance asset, shifting the policy problem from migration volume management to the creation of structural conditions that secure settlement through public service contribution. | Current framing: migration as a problem of national control requiring reduction through tighter eligibility and extended qualifying periods. Alternative: sustained public service contribution as a governance asset generating settlement entitlement, with immigration policy aligned to workforce sustainability rather than net migration reduction targets. | Such reframing would require the governance logic of immigration policy and workforce planning to be brought into alignment, displacing the current policy convergence between self-sufficiency signalling and settlement extension that compounds conditional permanence and may weaken the conditions supporting long-term professional belonging among internationally educated NHS nurses. |

**D6. NHS Pastoral Care Quality Award (NHS England, 2022–)**

***Governance Layer: Organisational Integration | Table 2, Row 7***

The NHS Pastoral Care Quality Award provides a framework for NHS organisations to demonstrate structured pastoral support for internationally educated nurses and midwives, establishing standards for induction, cultural orientation, buddying systems, and ongoing welfare support. Read through the WPR framework, the Award constructs international nurse integration as an organisational support problem requiring standardisation, positioning inclusion as an employer-delivered service rather than a governance entitlement and locating responsibility for integration at the organisational level rather than at the level of the governance conditions generating integration pressures.

| **WPR Question** | **Discursive Representation** | **Indicative Evidence** | **Analytical Implications for IEN Professional Identity** |
| --- | --- | --- | --- |
| **Q1: What is the problem represented to be?** | International nurse integration is positioned as a transitional support challenge requiring standardised organisational responses; the problem is located at the level of individual nurse wellbeing and employer support capacity. | Award criteria covering induction, cultural orientation, buddy systems, welfare support, and post-arrival monitoring. | Transitional Support Framing: By constructing integration as a support problem with a defined transitional arc, the Award implicitly positions the integration challenge as resolvable through organisational investment, potentially obscuring the structural governance conditions that extend beyond the transitional period. |
| **Q2: What assumptions underlie this representation?** | Integration challenges are assumed to be primarily transitional and amenable to structured organisational support; employer investment in pastoral care is assumed sufficient to address the professional identity challenges internationally educated nurses face. | Award criteria focus on the initial arrival period; pastoral support framed as a time-limited transitional intervention. | Transitional Assumption: The temporal framing of pastoral support as a transitional intervention reproduces the assumption that integration challenges resolve with time and employer support, without engaging with structural conditions that generate conditional belonging beyond the initial transition. |
| **Q3: How has this representation come about?** | Post-pandemic NHS recruitment surge generating large cohorts of newly arrived internationally educated nurses; high-profile cases of inadequate support; NMC concerns about fitness to practise implications of poor induction. | Introduced 2022 following NHS international recruitment expansion; linked to NMC Test of Competence requirements and employer-supervised pre-registration candidacy; associated with People Plan retention commitments. | Reactive Governance: The Award emerged in response to identified support failures rather than as a proactive governance framework, positioning pastoral care as a corrective response to recruitment-driven integration pressures. |
| **Q4: What is left unproblematised or silent?** | The Award does not address career progression beyond initial integration, seniority recognition, or the long-term belonging of internationally educated nurses beyond the transitional period it governs. | No criteria addressing career development pathways, seniority recognition, or medium-term professional identity support beyond initial arrival. | Post-Transition Silence: The Award’s focus on initial integration leaves the medium and long-term professional identity challenges of internationally educated nurses outside its governance scope, reproducing the pattern of addressing immediate operational pressures without structural engagement. |
| **Q5: What discursive effects are produced?** | Integration is constructed as an employer-delivered service for which NHS organisations bear primary responsibility; internationally educated nurses are positioned as recipients of support rather than contributors whose prior expertise warrants recognition. | Award language centres on employer support provision and nurse welfare; professional expertise recognition is not a criterion. | Recipient Positioning: The pastoral care governance discourse positions internationally educated nurses as beneficiaries of organisational support rather than professionals whose expertise warrants active recognition, potentially reinforcing the adaptive conformity dynamics documented in the qualitative evidence. |
| **Q6: How could the problem be represented differently?** | International nurse integration framed as a mutual professional development process in which employer organisations are required to actively recognise and deploy prior expertise, with Award criteria extending beyond transitional welfare to encompass medium-term career progression and professional recognition. | Current framing: employer-delivered transitional support. Alternative: mutual professional integration framework incorporating expertise recognition and career development criteria alongside pastoral care. | Such reframing would reposition IENs as professional contributors whose expertise requires active organisational recognition rather than transitional recipients of welfare support. |

**D7. Stay and Thrive Programme (NHS England, n.d)**

***Governance Layer: Retention Strategy | Table 2, Row 8***

The Stay and Thrive Programme provides nationally scaled guidance and resources to support the integration and retention of internationally educated nurses in NHS employment, offering toolkits, peer support frameworks, and workforce planning guidance to NHS organisations. Read through the WPR framework, the Programme constructs retention as an organisational challenge requiring national support infrastructure, positioning departure prevention as the primary governance objective and locating the explanation for attrition at the organisational level rather than in the structural governance conditions generating conditional belonging.

| **WPR Question** | **Discursive Representation** | **Indicative Evidence** | **Analytical Implications for IEN Professional Identity** |
| --- | --- | --- | --- |
| **Q1: What is the problem represented to be?** | IEN attrition is positioned as an organisational cost and workforce sustainability problem requiring national-level retention infrastructure; departure is constructed as the primary risk to be managed. | Retention-focused toolkit; turnover reduction framing; workforce stability as primary outcome measure. | Retention as Control: By constructing the problem as attrition rather than conditional belonging, the Programme frames the governance objective as keeping internationally educated nurses in post rather than ensuring genuine professional inclusion, potentially conflating physical presence with belonging. |
| **Q2: What assumptions underlie this representation?** | IEN departure is assumed to be preventable through enhanced organisational support; retention is assumed achievable without structural changes to the governance conditions generating conditional belonging. | Programme guidance emphasises employer action; no engagement with immigration policy or regulatory architecture as retention factors. | Organisational Sufficiency Assumption: The Programme assumes organisational-level retention interventions are sufficient to address departure risk, without engaging with the structural governance conditions — visa conditionality, regulatory verification, and self-sufficiency signalling — that may generate the belonging insecurity associated with attrition. |
| **Q3: How has this representation come about?** | High IEN attrition rates documented in NHS workforce data; investment in international recruitment generating organisational pressure to recover costs through retention; NHS People Plan retention commitments. | Post-pandemic IEN recruitment costs; NMC registration data showing IEN departure rates; NHS England retention strategy development. | Cost Recovery Logic: The Programme emerged partly from the need to recover significant international recruitment investment, positioning retention as an organisational financial imperative alongside a workforce sustainability objective. |
| **Q4: What is left unproblematised or silent?** | The Programme does not address the structural governance conditions generating departure risk: employment-contingent visa residency, extended ILR qualifying periods, self-sufficiency signalling, or the absence of career progression pathways. | No engagement with immigration policy reform; no governance recommendation regarding LTWP self-sufficiency signals; no career progression framework. | Structural Silence: By focusing on organisational retention practice without engaging with the governance conditions generating conditional belonging, the Programme reproduces the downstream positioning of integration initiatives identified across the governance architecture. |
| **Q5: What discursive effects are produced?** | Internationally educated nurses are positioned as potential leavers requiring organisational retention management rather than as professionals whose long-term commitment depends on structural governance conditions beyond employer control. | Toolkit language frames IENs as at-risk of departure; employer action plans as primary governance response; IEN agency in departure decisions not foregrounded. | Deficit Positioning: The retention discourse positions internationally educated nurses as requiring management to prevent departure, potentially reinforcing the perception of their presence as conditional and their commitment as uncertain rather than stable. |
| **Q6: How could the problem be represented differently?** | IEN retention framed as a governance outcome dependent on structural conditions across immigration, regulatory, and workforce policy domains, with national-level accountability for the governance architecture generating departure risk alongside organisational support guidance. | Current framing: organisational retention management. Alternative: multilevel governance accountability for structural conditions generating conditional belonging, with organisational support as one component of a broader governance response. | Such reframing would locate the primary governance responsibility for IEN retention at the level of national policy architecture rather than exclusively at the organisational level, more accurately reflecting the structural conditions generating departure risk. |

***Note.*** *WPR = What’s the Problem Represented to Be? IEN = internationally educated nurse. NHS = National Health Service. LTWP = NHS Long Term Workforce Plan. NMC = Nursing and Midwifery Council. WRES = Workforce Race Equality Standard. ILR = indefinite leave to remain. BME = Black and Minority Ethnic. CBT = computer-based test. OSCE = objective structured clinical examination. Documents D1–D7 correspond to Table 2 Rows 2–8 in the main manuscript. The NHS Long Term Workforce Plan (Table 2, Row 1) is analysed separately in Table 1 of the main manuscript and is not reproduced here.*
