# Supplementary material for: The Paradox of Workforce Self‐Sufficiency: Policy Narratives, Professional Identity and Internationally Educated Nurses in the United Kingdom
Source: Nurs Inq. 2026 Sep 8;33(4):e70164. doi: 10.1111/nin.70164 (PMC13554827; doi:10.1111/nin.70164)
Supplement: Supplementary file 2 [file NIN-33-e70164-s002.docx]

**Supplementary File 1.**

This flow diagram presents the study selection process for the qualitative evidence synthesis strand of this review, following the PRISMA 2020 reporting guidelines (Page et al., 2021). The search was conducted across four peer-reviewed databases: CINAHL, MEDLINE, Scopus, and Web of Science.

**Supplementary Figure 1. PRISMA 2020 Flow Diagram of Study Selection**

Note. PRISMA = Preferred Reporting Items for Systematic Reviews and Meta-Analyses. IEN = internationally educated nurse. Exclusion reasons at full-text stage reflect application of the eligibility criteria described in Section 3.3. The search was conducted between January 7-10, 2026. Grey literature was not included in the qualitative evidence search; targeted organisational searches for policy documents are described in Section 3.2.
