# Supplementary material for: The Paradox of Workforce Self‐Sufficiency: Policy Narratives, Professional Identity and Internationally Educated Nurses in the United Kingdom
Source: Nurs Inq. 2026 Sep 8;33(4):e70164. doi: 10.1111/nin.70164 (PMC13554827; doi:10.1111/nin.70164)
Supplement: Supplementary file 3 [file NIN-33-e70164-s003.docx]

**Supplementary File 2**

*Study Characteristics and Critical Appraisal of Included Studies*

**Supplementary Table S1. *Characteristics of Included Studies (N = 17)***

| **#** | **Author(s) (Year)** | **Study Design** | **Sample** | **Country of Origin** | **UK Setting** | **Thematic Focus** | **JBI Score** | **Primary Construct** |
| --- | --- | --- | --- | --- | --- | --- | --- | --- |
| 1 | Okougha & Tilki (2010) | Qualitative: Grounded theory; focus groups | n=13 | Ghana; Philippines | NHS London | Acculturation stress; communication barriers; misrecognition of competence | 9.5/10 | Mediated professional Legitimacy  Identity Renegotiation |
| 2 | Batnitzky & McDowell (2011) | Qualitative: In-depth interviews | n=21 | Caribbean; South; Asia, East Africa | NHS UK cities | Institutional discrimination; racialisation; emotional labour | 9.0/10 | Identity Renegotiation |
| 3 | Alexis & Shillingford (2012) | Qualitative: Semi-structured interviews | n=13 | Jamaica; Philippines | NHS London | Regulatory navigation; restricted autonomy; delayed recognition | 10/10 | Mediated Professional Legitimacy |
| 4 | Adhikari (2013) | Qualitative: Multi-sited ethnography; In-depth Interview | n=21 | Nepal | NHS UK | Gender dynamics; family migration; economic empowerment | 10/10 | Identity Renegotiation |
| 5 | Alexis (2013) | Qualitative: Hermeneutic phenomenology; Interview and focus group | n=36 | Philippines; South Africa; Caribbean, Sub-Saharan Africa | NHS England | Adaptive conformity; hybrid identity; marginalisation | 10/10 | Identity Renegotiation; Mediated Professional Legitimacy |
| 6 | Likupe (2013) | Qualitative: Focus groups and interviews | n=30 | Sub-Saharan Africa | NHS UK | Brain drain; skills underutilisation; professional marginalisation | 9.0/10 | Identity Renegotiation |
| 7 | Bidwell et al. (2014) | Qualitative: Content analysis; interviews | n=10 | South Africa | NHS UK | Employment security; visa-linked insecurity; migration rationale | 8.5/10 | Conditional Belonging |
| 8 | Adhikari & Melia (2015) | Qualitative: Multi-sited ethnography | n=21 | Nepal | NHS UK | Workforce mismanagement; visa sponsorship dependency | 10/10 | Mediated Professional Legitimacy; Conditional Belonging |
| 9 | Al-Hamdan et al. (2015) | Qualitative: Individual interviews | n=25 | Jordan | NHS UK | Professional transformation; identity reconstruction; belonging | 9.0/10 | Identity Renegotiation; Mediated Professional Legitimacy |
| 10 | Alexis & Shillingford (2015) | Qualitative: Semi-structured interviews | n=13 | Jamaica; Philippines | NHS London | Revalidation experiences; re-establishment of professional confidence | 10/10 | Mediated Professional Legitimacy |
| 11 | Likupe (2015) | Qualitative: Focus groups and interviews | n=30 | Africa (sub-Saharan) | NHS UK | Racism; everyday discrimination; racialised workplace culture | 10/10 | Identity Renegotiation |
| 12 | Stubbs (2017) | Qualitative descriptive: In-depth interviews | n=16 | India | NHS (ONP) | ONP; supervised practice; credentialling process | 7.5/10 | Mediated Professional Legitimacy |
| 13 | Smith & Gillin (2021) | Qualitative: Interviews and focus groups | n=24 | Philippines | NHS UK | Ontological security; migration choice; stability-seeking | 10/10 | Conditional Belonging; Identity Renegotiation |
| 14 | Spiliopoulos & Timmons (2023) | Qualitative: Semi-structured interviews | n=15 | Multiple (EU and non-EU) | NHS UK | Post-Brexit precarity; tolerated citizenship; belonging | 10/10 | Conditional Belonging |
| 15 | Pressley et al. (2023) | Mixed methods: Online survey | n=655 | Multiple (globally recruited) | NHS England | Initial integration; support structures; retention | 10/10 | Identity Renegotiation; Mediated Professional Legitimacy |
| 16 | Ugiagbe (2024) | Qualitative: IPA | n=8 | Nigeria | NHS UK | Career progression; racialised barriers; ontological security | 10/10 | Conditional Belonging |
| 17 | Pressley et al. (2025) | Mixed methods: Exploratory survey | n=773 | Multiple (globally recruited) | NHS England | Two-year integration; retention; workplace belonging | 9.5/10 | Identity Renegotiation; Mediated Professional Legitimacy |

*Note. IEN = internationally educated nurse; NHS = National Health Service; ONP = Overseas Nurses Programme; IPA = interpretative phenomenological analysis. Studies are ordered chronologically by year of publication. JBI Score reflects the Joanna Briggs Institute Critical Appraisal Checklist for Qualitative Research (out of 10); criterion-level appraisal is provided in Supplementary Table S2. The Primary Construct column reflects the analytic categorisation derived through the thematic synthesis described in Section 3.5. Several studies address cross-cutting themes across multiple categories. All studies are UK-based and peer-reviewed. For the two mixed-methods studies (Pressley et al., 2023, 2025), only the qualitative findings were extracted and synthesised; these strands were appraised using the JBI qualitative checklist, consistent with the qualitative focus of this synthesis.*

**Supplementary Table S2. *JBI Critical Appraisal Checklist Scores for Included Studies (N = 17)***

Appraisal was conducted using the Joanna Briggs Institute Critical Appraisal Checklist for Qualitative Research (Joanna Briggs Institute, 2020), comprising ten criteria (C1–C10). Each criterion was rated Yes (Y), No (N), or Unclear (U).

| **Study** | **C1** | **C2** | **C3** | **C4** | **C5** | **C6** | **C7** | **C8** | **C9** | **C10** | **Total** | **Tier** |
| --- | --- | --- | --- | --- | --- | --- | --- | --- | --- | --- | --- | --- |
| Okougha & Tilki (2010) | Y | Y | Y | Y | Y | Y | U | Y | Y | Y | **9.5** | High |
| Batnitzky & McDowell (2011) | Y | Y | Y | Y | Y | U | U | Y | Y | Y | **9.0** | High |
| Alexis & Shillingford (2012) | Y | Y | Y | Y | Y | Y | Y | Y | Y | Y | **10.0** | High |
| Adhikari (2013) | Y | Y | Y | Y | Y | Y | Y | Y | Y | Y | **10.0** | High |
| Alexis (2013) | Y | Y | Y | Y | Y | Y | Y | Y | Y | Y | **10.0** | High |
| Likupe (2013) | Y | Y | Y | Y | Y | U | U | Y | Y | Y | **9.0** | High |
| Bidwell et al. (2014) | Y | Y | Y | Y | Y | U | U | U | Y | Y | **8.5** | High |
| Adhikari & Melia (2015) | Y | Y | Y | Y | Y | Y | Y | Y | Y | Y | **10.0** | High |
| Al-Hamdan et al. (2015) | Y | Y | Y | Y | Y | U | U | Y | Y | Y | **9.0** | High |
| Alexis & Shillingford (2015) | Y | Y | Y | Y | Y | Y | Y | Y | Y | Y | **10.0** | High |
| Likupe (2015) | Y | Y | Y | Y | Y | Y | Y | Y | Y | Y | **10.0** | High |
| Stubbs (2017) | U | Y | Y | Y | Y | N | N | Y | Y | Y | **7.5** | High |
| Smith & Gillin (2021) | Y | Y | Y | Y | Y | Y | Y | Y | Y | Y | **10.0** | High |
| Spiliopoulos & Timmons (2023) | Y | Y | Y | Y | Y | Y | Y | Y | Y | Y | **10.0** | High |
| Pressley et al. (2023) | Y | Y | Y | Y | Y | Y | Y | Y | Y | Y | **10.0** | High |
| Ugiagbe (2024) | Y | Y | Y | Y | Y | Y | Y | Y | Y | Y | **10.0** | High |
| Pressley et al. (2025) | Y | Y | Y | Y | Y | Y | U | Y | Y | Y | **9.5** | High |

*Note. Y = Yes (1); N = No (0); U = Unclear (0.5). C1–C10 correspond to the ten items of the JBI Critical Appraisal Checklist for Qualitative Research (Joanna Briggs Institute, 2020). Total reflects the number of criteria rated Yes out of 10. All included studies scored 7.5 or above, consistent with the quality threshold applied in the synthesis. For the two mixed-methods studies (Pressley et al., 2023, 2025), only the qualitative component was appraised, consistent with the qualitative focus of this synthesis.*
