# Supplementary material for: The Paradox of Workforce Self‐Sufficiency: Policy Narratives, Professional Identity and Internationally Educated Nurses in the United Kingdom
Source: Nurs Inq. 2026 Sep 8;33(4):e70164. doi: 10.1111/nin.70164 (PMC13554827; doi:10.1111/nin.70164)
Supplement: Supplementary file 4 [file NIN-33-e70164-s004.docx]

**Supplementary File 4**

**Thematic Synthesis Matrix: Internationally Educated Nurses’ Professional Experience in the NHS**

*Developed using Thomas and Harden (2008) thematic synthesis approach | n = 17 studies*

*Theoretical sensitisation: Social Identity Theory (Tajfel & Turner, 1979); Ontological Security (Giddens, 1991)*

**Methodological Note**

This matrix presents the thematic synthesis of 17 qualitative studies examining internationally educated nurses’ (IENs) professional experience within the NHS, conducted following the three-stage approach described by Thomas and Harden (2008):

• Stage 1 – Free line-by-line coding: Findings and conclusions of each included study were coded inductively, generating initial descriptive codes close to the data.

• Stage 2 – Development of descriptive themes: Initial codes were grouped into descriptive themes reflecting recurrent patterns across studies without departing from the primary study data.

• Stage 3 – Generation of synthetic constructs: Descriptive themes were interpreted through Social Identity Theory and Ontological Security to generate synthetic constructs that go beyond the primary studies to address the review question.

The matrix below presents the three analytical themes produced by this process, alongside their descriptive sub-themes, first-order line-by-line codes, descriptive characteristics synthesised across studies, contributing studies, and interpretive theoretical lenses. The ‘Analytical Claim: Critical Interrogation of Primary Framings’ column represents Stage 3 output: interpretive claims that go beyond the data to address how governance narratives shape professional identity, and documents where primary study framings were critically interrogated, reworked, or reconceptualised through analytic dialogue between the two strands, consistent with the critical interpretive orientation of CQES (critical qualitative evidence synthesis) (Dixon-Woods et al., 2006; Flemming et al., 2019).

| **Higher-Order Synthetic Construct** | **Descriptive Sub-Theme** | **First-Order Codes (Stage 1)** | **Descriptive Theme (Stage 2)** | **Analytical Claim: Critical Interrogation of Primary Framings (Stage 3)** | **Contributing Studies (n=17)** | **Interpretive Lens** |
| --- | --- | --- | --- | --- | --- | --- |
| **Mediated Professional Legitimacy** | **Status Disruption and Professional Repositioning** | **Entry-level**  • Regulatory verification as gatekeeping  • Supervised practice despite prior seniority  • Band 5 entry regardless of prior Band 7+ experience  • Competency re-testing of demonstrated skills | Regulatory processes systematically reposition IENs below prior levels of professional authority during NHS entry, irrespective of prior seniority or specialist expertise. Competence assurance mechanisms are experienced as institutional revalidation rather than professional development. | Status disruption appears not incidental to NHS integration but a recurrent outcome of a regulatory architecture that constructs internationally acquired expertise as requiring domestic verification before professional legitimacy can be accorded. This governance logic, visible in both the ONP era and the current CBT/OSCE pathway, produces a period of enforced professional subordination that reflects the conditions of regulatory design rather than individual deficiency. | • Stubbs (2017)  • Alexis & Shillingford (2012; 2015)  • Adhikari & Melia (2015)  • Okougha & Tilki (2010)  • Al-Hamdan et al. (2015)  • Alexis (2013)  • Pressley et al. (2023; 2025) | • Social Identity Theory  • Ontological Security (professional continuity disruption) |
|  | **Mediated Legitimacy through Institutional and Regulatory Validation** | **Institutional**  • Employer-mediated professional recognition  • Legitimacy contingent on organisational validation  • Supervisory hierarchies constraining clinical voice  • Misrecognition of competence by colleagues | Professional legitimacy for IENs requires mediation through institutional and regulatory validation rather than being accorded automatically on the basis of qualifications and experience. Organisational and regulatory processes function as gatekeepers determining the conditions under which prior expertise is recognised. | The mediation of professional legitimacy through domestic institutional processes reflects a governance logic that positions internationally acquired expertise as supplementary to the domestic training pathway, requiring organisational endorsement before full professional authority is accorded. This dynamic may not be fully addressable through individual adaptation alone; it appears embedded in the regulatory and organisational architecture of NHS entry. | • Alexis & Shillingford (2012; 2015)  • Okougha & Tilki (2010)  • Stubbs (2017)  • Adhikari & Melia (2015)  • Al-Hamdan et al. (2015)  • Alexis (2013) | • Social Identity Theory (status differentiation; group positioning) |
| **Identity Renegotiation** | **Adaptive Reconstruction** | **Adaptive strategies**  • Communication style modification  • Pursuit of locally recognised qualifications  • Selective assimilation into dominant professional culture  • Suppression of prior clinical knowledge  • Cultural competence performance on organisation’s terms | IENs engage in systematic modification of professional self-presentation to demonstrate competence within domestically defined norms. Adaptive strategies are consistently framed as resilience within policy and organisational discourse, obscuring their asymmetric character. | Adaptive reconstruction is not evidence of successful integration but a response to asymmetric institutional conditions in which the terms of professional legitimacy are defined by the dominant domestic workforce pathway rather than by internationally acquired expertise. The burden of reconstruction falls disproportionately on internationally educated nurses rather than on the institutional cultures receiving them, suggestive of a governance environment that constructs adaptation as an IEN responsibility. | • Batnitzky & McDowell (2011)  • Alexis (2013)  • Likupe (2013; 2015)  • Adhikari (2013)  • Pressley et al. (2023; 2025)  • Al-Hamdan et al. (2015)  • Okougha & Tilki (2010) | • Social Identity Theory (boundary construction; inclusion vs. exclusion dynamics) |
|  | **Hybrid Identity Formation** | **Hybrid identity**  • Preservation of prior expertise in non-visible form  • Development of dual professional identity  • Cultural knowledge maintained alongside adaptive conformity  • Neither fully visible nor fully abandoned prior self | Rather than wholesale assimilation or rejection of professional norms, IENs develop hybrid professional identities that preserve prior expertise and cultural knowledge alongside adaptive conformity to dominant clinical expectations. Hybrid identity formation represents a creative response to asymmetric institutional conditions rather than authentic professional integration. | Hybrid identity formation reflects the conditions of a discursive environment that renders internationally acquired expertise invisible unless domestically validated. IENs preserve prior professional selves in forms that cannot be officially recognised but are not abandoned. This dual-identity dynamic has implications for professional continuity and long-term retention that organisational integration programmes, focused on adaptation rather than recognition, may have limited capacity to address. | • Alexis (2013)  • Batnitzky & McDowell (2011)  • Al-Hamdan et al. (2015)  • Adhikari (2013)  • Pressley et al. (2023; 2025) | • Social Identity Theory (group positioning; status differentiation)  • Ontological Security (continuity of professional self) |
| **Conditional Belonging** | **Negotiated Belonging** | **Material conditions**  • Visa sponsorship dependency  • Employment-contingent settlement rights  • Residency tied to sponsored employer  • Settlement uncertainty during ILR qualifying period | IENs experience belonging as conditional on continued employment, regulatory status, and institutional goodwill rather than as an unconditional feature of professional membership. Present operational indispensability does not translate into a sense of future security or permanence within the NHS workforce. | Conditional belonging may be less a perception requiring correction through better organisational communication than an interpretively grounded reading of a governance environment that is, in material and symbolic terms, experienced as conditional. Internationally educated nurses report not uncertainty about whether they are valued — many accounts confirm they are — but uncertainty about whether that value will persist and on whose terms. This distinction is analytically significant: it locates the problem in governance conditions rather than individual perception or organisational communication. | • Smith & Gillin (2021)  • Spiliopoulos & Timmons (2023)  • Bidwell et al. (2014)  • Ugiagbe (2024)  • Adhikari & Melia (2015)  • Pressley et al. (2023; 2025) | • Ontological Security (conditional permanence; employment-contingent security)  • Social Identity Theory (inclusion vs. exclusion dynamics) |
|  | **Conditional Permanence** | **Stability-seeking**  • Migration motivated by ontological security-seeking  • Family welfare and settlement as primary driver  • Professional stability sought through NHS employment  • Gap between aspiration and structural reality | IENs’ migration decisions are motivated by ontological security-seeking, the desire for professional and personal stability, yet they encounter a governance environment that structurally undermines the conditions for that stability. Governance signals from multiple policy instruments converge to construct IEN presence as provisional, intensifying rather than resolving the insecurity that motivated migration. | When the governance horizon within which internationally educated nurses must plan their professional futures remains uncertain, the capacity to maintain a coherent and continuous professional self may be less a matter of individual resilience than of the governance conditions within which professional life is structured. When immigration policy, workforce planning signals, and regulatory requirements collectively narrow the conditions of predictability upon which stable professional identity depends, the disruption may not resolve through time and familiarity but persist through the structural conditions within which professional life continues to be lived. | • Smith & Gillin (2021)  • Spiliopoulos & Timmons (2023)  • Bidwell et al. (2014)  • Ugiagbe (2024)  • Adhikari & Melia (2015) | • Ontological Security (predictability of social environment; future stability)  • Social Identity Theory (conditional workforce positioning) |

*Note. IEN = internationally educated nurse. NHS = National Health Service. ONP = Overseas Nurses Programme. CBT = computer-based test. OSCE = objective structured clinical examination. ILR = indefinite leave to remain. Themes are numbered 1 (Mediated Professional Legitimacy), 2 (Identity Renegotiation) and 3 (Conditional Belonging) throughout. Study identifiers in the Contributing Studies column correspond to those in Supplementary Table S1 (Supplementary File 2), where full criterion-level JBI appraisal scores are also presented; within each cell, studies are listed by analytic prominence rather than in Table S1 order. Alexis & Shillingford (2012, 2015), Adhikari (2013) and Adhikari & Melia (2015), and Likupe (2013, 2015) report on linked participant samples. Each appears as a separate study in Table S1 and was appraised independently, reflecting its distinct analytic focus; in this synthesis they are cited as paired reports to avoid overstating the independence of recurrent patterns.*

**Cross-Cutting Analytical Patterns**

The following patterns were identified as recurrent across all three analytical themes and across the full 15-year review period:

• Structural rather than incidental explanation: The recurrence of similar identity dynamics across fifteen years of evidence, diverse national origins (Africa, South and Southeast Asia, Caribbean, Middle East, EU and non-EU), and varied NHS clinical settings warrants a structural rather than incidental analytical account. Individual variation in outcome does not dissolve the pattern; where conditions of recognition, progression, and stability were present, nurses achieved what the governance architecture otherwise makes difficult to achieve.

• Asymmetry of integration: Across all three themes, the burden of adjustment falls consistently on internationally educated nurses rather than on the institutional cultures, regulatory systems, or governance frameworks receiving them. This asymmetry reflects a governance configuration that positions domestic training as the normative pathway and international recruitment as a managed departure from that norm.

• Conditionality as persistent feature not transitional phase: Evidence from studies spanning 2010 to 2025 consistently documents that conditional belonging, mediated legitimacy, and identity renegotiation do not resolve with time, familiarity, or organisational support. They are reproduced through the structural conditions within which professional life continues to be lived, suggesting that the explanation lies in governance conditions rather than integration programme adequacy.

• Interconnection of the three dynamics: Status disruption, adaptive reconstruction, and conditional belonging are not separate phenomena but interconnected expressions of a single governance logic that constructs internationally educated nurses as conditionally present within a workforce that structurally depends on their permanence. The paradox of self-sufficiency is not only a policy-level construct; it is lived at the level of professional identity.

**Studies Contributing to Multiple Themes**

Several studies contribute to more than one analytical theme, reflecting the cross-cutting nature of the identity dynamics documented. The following studies contribute to at least two of the three higher-order synthetic constructs:

- Okougha & Tilki (2010): Themes 1 and 2
- Alexis (2013): Themes 1 and 2
- Al-Hamdan et al. (2015): Themes 1 and 2
- Adhikari & Melia (2015): Themes 1 and 3
- Pressley et al. (2023; 2025): Themes 1, 2 and 3
